# The *yjdF* riboswitch candidate regulates gene expression by binding diverse azaaromatic compounds

**DOI:** 10.1261/rna.054890.115

**Published:** 2016-04

**Authors:** Sanshu Li, Xue Ying Hwang, Shira Stav, Ronald R. Breaker

**Affiliations:** 1 Howard Hughes Medical Institute, Yale University, New Haven, Connecticut 06520-8103, USA; 2 Department of Molecular, Cellular and Developmental Biology, Yale University, New Haven, Connecticut 06520-8103, USA; 3 Department of Molecular Biophysics and Biochemistry, Yale University, New Haven, Connecticut 06520-8103, USA

**Keywords:** alkaloid, chelerythrine, DUF2992, noncoding RNA, orphan riboswitch

## Abstract

The *yjdF* motif RNA is an orphan riboswitch candidate that almost exclusively associates with the *yjdF* protein-coding gene in many bacteria. The function of the YjdF protein is unknown, which has made speculation regarding the natural ligand for this putative riboswitch unusually challenging. By using a structure-probing assay for ligand binding, we found that a surprisingly broad diversity of nitrogen-containing aromatic heterocycles, or “azaaromatics,” trigger near-identical changes in the structures adopted by representative *yjdF* motif RNAs. Regions of the RNA that undergo ligand-induced structural modulation reside primarily in portions of the putative aptamer region that are highly conserved in nucleotide sequence, as is typical for riboswitches. Some azaaromatic molecules are bound by the RNA with nanomolar dissociation constants, and a subset of these ligands activate riboswitch-mediated gene expression in cells. Furthermore, genetic elements most commonly adjacent to the *yjdF* motif RNA or to the *yjdF* protein-coding region are homologous to protein regulators implicated in mitigating the toxic effects of diverse phenolic acids or polycyclic compounds. Although the precise type of natural ligand sensed by *yjdF* motif RNAs remains unknown, our findings suggest that this riboswitch class might serve as part of a genetic response system to toxic or signaling compounds with chemical structures similar to azaaromatics.

## INTRODUCTION

Over 30 distinct riboswitch classes have been discovered that sense and respond to numerous metabolites and ions (Zhang et al. 2010; Breaker 2011; Serganov and Nudler 2013). Among the most common riboswitch classes are those that respond to ligands serving fundamental roles in the metabolism of all organisms, including numerous coenzymes, amino acids, and nucleotide derivatives. Each of these novel riboswitch classes provides opportunities to establish new pathways for gene regulation. Moreover, members of each riboswitch class can serve as model systems to reveal, at atomic resolution, how RNAs can form selective receptors for their natural ligands, and how ligand binding is translated into gene regulation events (Garst et al. 2011; Serganov and Patel 2012).

Surprisingly, several common riboswitch classes sense molecules or other ligands whose roles and gene regulation activities have been less well understood, despite the widespread biological importance of these ligands. For example, there are now three distinct classes of riboswitches that sense the nucleobase derivative called pre-queuosine 1 (preQ_1_) (McCown et al. 2014). PreQ_1_ is a modified base present in organisms from at least two domains of life whose functions are only incompletely established. Despite the relative obscurity of preQ_1_, the widespread distribution of members of these three riboswitch classes strongly suggests that the compound is near ubiquitous in bacteria and performs critical roles in many species. Similarly, the discovery of fluoride riboswitches in many members of two domains of life (Baker et al. 2012) has revealed a large collection of genes whose functions are related to overcoming fluoride toxicity. Without knowledge of this fluoride-binding RNA, the functions of many of these genes would have remained mysterious.

As a result, the ongoing search for more riboswitch classes promises to reveal additional ligands for riboswitches and expose deeper understanding of their roles in biology. Specifically, once a riboswitch class has been experimentally validated, bioinformatics methods can be used both to better appreciate the regulatory role its cognate ligand performs in cells and to link the function of proteins to this ligand. For example, the widespread importance of the bacterial signaling dinucleotides c-di-AMP and c-AMP-GMP was quickly appreciated in part because of the discovery of novel riboswitch classes that sense these two compounds (Nelson et al. 2013, 2015; Kellenberger et al. 2015; Ren et al. 2015).

Perhaps the greatest opportunities for generating novel understanding regarding the signaling functions of riboswitch ligands are associated with “orphan” riboswitch candidates. These orphans are RNA motifs believed to be riboswitches, but whose ligands remain to be discovered despite much experimental effort and the passage of time. There are several possible reasons why orphan riboswitch classes resist experimental resolution. For example, their ligands might have been unknown to scientists at the time of the discovery of the RNA motif, as was the case for c-di-AMP riboswitches (Barrick et al. 2004; Block et al. 2010; Nelson et al. 2013). Some natural ligands had been considered to be relatively unimportant to biology, or at least were not considered by many to be important for regulating gene expression, as was the case for fluoride (Baker et al. 2012) and for ZTP (Kim et al. 2015) riboswitches. In other instances, the biochemical systems that manage the concentration of the ligand were not well established, as was the case for Mn^2+^ riboswitches (Barrick et al. 2004; Meyer et al. 2011; Dambach et al. 2015; Price et al. 2015) and for all the other riboswitch ligands noted immediately above.

Moreover, one of the best indicators of riboswitch ligand specificity comes from gene associations, as it is frequently obvious what ligand would be most reasonable to regulate the expression of a gene in a metabolic process or toxin response pathway. Unfortunately, riboswitch candidates that are very rare provide only a few examples of genes from which to establish gene associations. Even a common orphan riboswitch, with hundreds of representatives, can have a genetic context that is devoid of clues regarding ligand identity. This is particularly true when the genes most commonly associated with a riboswitch candidate codes for proteins whose functions are unknown.

**FIGURE 1.**
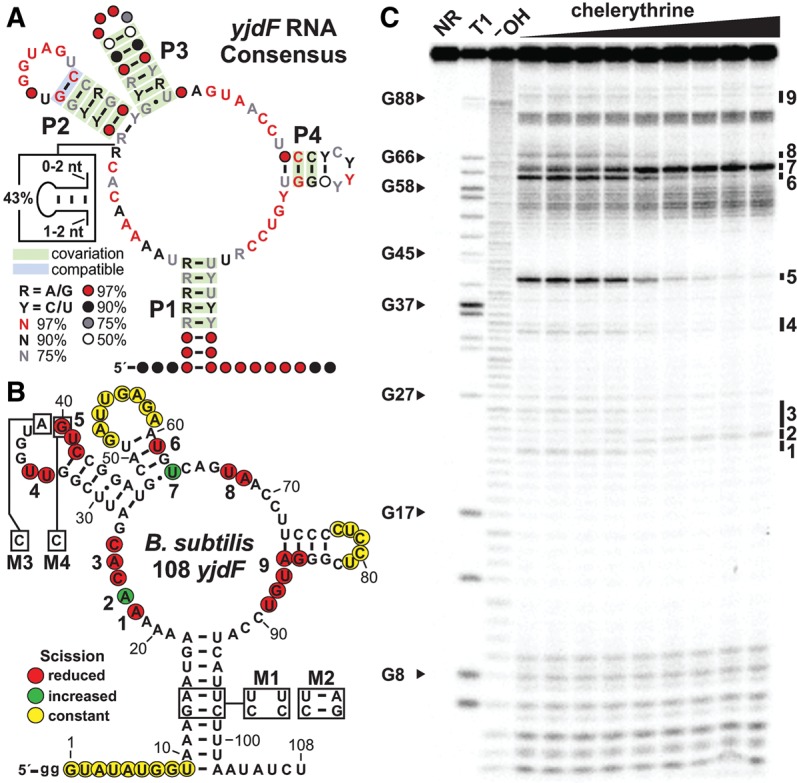
*yjdF* motif RNAs exhibit biochemical characteristics of riboswitch aptamers. (*A*) Consensus sequence and structure of *yjdF* motif RNAs based on 1060 distinct RNA representatives. Base-paired substructures P1 through P4, and sometimes an additional variable-sequence stem (box), are interspersed among regions of high sequence conservation. The percentage of representatives carrying the nucleotides depicted are indicated by colored letters (R is G or A, Y is C or U) or circles (designating the presence of any nucleotide), and colored boxes indicate there is evidence for natural sequence covariation or compatible mutation that retains base-pairing. (*B*) Sequence and secondary structure of the 108 *yjdF* RNA construct based on the 5′ UTR of the *yjdF* gene from *Bacillus subtilis*. The nucleotides altered in mutated RNA constructs M1, M2, M3, and M4 are depicted in boxes. Regions that undergo RNA strand scission as revealed by the in-line probing data depicted in C are identified with colored circles based on their characteristics. Nine regions that undergo increased or decreased scission are numbers 1–9. (*C*) Polyacrylamide gel electrophoresis (PAGE) analysis of in-line probing reactions of the 5′ ^32^P-labeled 108 *yjdF* construct with concentrations of the natural alkaloid drug compound chelerythrine ranging from 0 to 300 nM. NR, T1, and ^−^OH designate no reaction, partial digestion with RNase T1, and partial digestion with alkali, respectively. Select bands corresponding to RNase T1 digestion after G residues are labeled according to the numbering system in *B*. Numbers 1–9 designate changes in banding patterns at the sites denoted in *B*.

This latter problem exists for an orphan riboswitch candidate called the *yjdF* motif (Weinberg et al. 2010), so named because it commonly resides in the 5′-untranslated region (UTR) of the protein-coding gene *yjdF* (or DUF2992). This RNA motif is characterized by its formation of a four-stem junction interspersed with regions of highly conserved nucleotides (Fig. 1A). Although we have identified 1060 distinct examples of this class, their near universal association with this single gene of unknown function (Supplemental Fig. S1) has precluded the rational pursuit of ligand validation studies. Therefore, we have used a dual strategy of formulating ligand hypotheses based on rare gene associations and testing a variety of diverse chemical compounds for possible binding by *yjdF* motif RNAs. Our results demonstrate that *yjdF* motif RNAs are riboswitches that can regulate gene expression by binding to an unusually large diversity of polycyclic aromatic nitrogen-containing heterocycles, which are sometimes called PANHs (Östman and Colmsjö 1987) or azaaromatics (Keefer and Johnson 1970; Zheng et al. 2010). We hypothesize that these “azaaromatic riboswitches” might have evolved to bind natural members of a class of large, planar, and hydrophobic compounds to activate production of the YjdF protein, perhaps as a mechanism for detoxification. If true, azaaromatic riboswitches would be analogous to protein receptors that bind diverse polycyclic aromatic hydrocarbon (PAH) compounds and activate gene expression to regulate natural PAH production or to overcome PAH toxicity (by activating multidrug resistance proteins) as seen in some bacterial species (Huillet et al. 2006; Lubelski et al. 2006).

## RESULTS AND DISCUSSION

### An updated consensus sequence and structural model of *yjdF* motif RNAs from an expanded collection of representatives

Previously, *yjdF* motif RNAs were found only in bacterial species classified as Firmicutes (Weinberg et al. 2010). Given the ongoing expansion in genomic sequence data, we conducted another search for additional representatives by using an RNA homology analysis algorithm called Infernal (Nawrocki and Eddy 2013) to search more recent DNA sequence databases (see Materials and Methods). A total of 1060 representatives were identified, wherein most are present in Firmicutes. However, examples were also identified in species of Actinobacteria, Fusobacteria, Spirochaetes, and Synergistetes. This large number of representatives and their distribution in more diverse species suggests that the natural ligand for this orphan riboswitch is also likely to be relatively prominent among many organisms.

The revised consensus sequence and structural model for the RNA motif is very similar to the previous model (Weinberg et al. 2010), with two exceptions. First, over 40% of the representatives carry an optional base-paired structure immediately preceding the P2 stem. When this additional hairpin is present, the P2 stem is usually formed by 5 base pairs (bp) rather than the typical 6 bp. Second, some nucleotides, especially in the P4 stem, are less conserved than previously depicted. This finding is consistent with our original hypothesis that nucleotides in this stem–loop substructure can form an alternative base-paired interaction with nucleotides of the adjoining gene's ribosome-binding site (RBS, see below). This arrangement is expected for an expression platform that controls translation via ligand-dependent modulation of ribosome-binding site access (Breaker 2011, 2012), which is an unusual mechanism for genetic regulation of riboswitches found predominantly in Firmicutes. If base-pairing between nucleotides in P4 and the RBS occurs only in the absence of ligand, then the riboswitch is expected to function as a genetic “ON” switch when ligand is bound.

### Unusual riboswitch ligand specificity revealed by compound screening

As with our previous efforts to identify the ligands sensed by riboswitch classes, we examined the gene that resides immediately downstream from 859 nonidentical *yjdF* orphan riboswitch representatives (Supplemental Fig. S1A). The vast majority of riboswitch examples appear to control a single open reading frame coding for the YjdF protein (also called DUF2992). Unfortunately, the function of this protein remains unknown, and homology searches reveal only modest similarity to other proteins. Notably, the C-terminal region of each protein representative is greatly enriched for lysine and arginine amino acids. On average within the last 35 amino acids, lysine and arginine comprise 43.3% of the amino acids, and representatives range from 57.1% to 28.5%. This abundance of positively charged sidechains is common with proteins that interact with polynucleotide chains. However, the function of this protein remains unknown, and we were unable to formulate strong hypotheses regarding the possible identity of the natural ligand by relying only on clues derived from the functions of adjoining genes. Therefore, we relied on two alternative approaches to search for the natural ligand.

For our first approach, we examined the in vitro binding characteristics of several coenzymes and coenzyme derivatives, including nicotinamide adenine dinucleotide (NAD) and flavin mononucleotide (FMN). These compounds were chosen for investigation because, in rare instances, several genes related to NAD and flavin metabolism are located near the *yjdF* gene (Supplemental Fig. S1B). Also, numerous riboswitch classes are already known that selectively respond to universally distributed nucleotide-like coenzymes (Breaker 2012). To detect binding, we used an assay called in-line probing (Soukup and Breaker 1999; Regulski and Breaker 2008). This assay reveals ligand-mediated shape changes in RNA structures upon binding by monitoring the spontaneous cleavage of RNA phosphodiester linkages. We observed that precise and reproducible changes in the banding patterns of a 108-nucleotide (nt) *yjdF* (108 *yjdF*) RNA construct derived from *Bacillus subtilis* (Fig. 1B) were produced by FMN, riboflavin, and numerous flavin analogs (data not shown). Surprisingly, even extremely distant analogs such as proflavine induced the same changes in the banding pattern revealed by in-line probing (Supplemental Fig. S2A). These results suggest that a diverse collection of compounds can be bound by the 108 *yjdF* RNA, and that the RNA undergoes the same global change in RNA folding regardless of the ligand bound.

The ligand-binding aptamer portion of riboswitches is commonly formed from the most-conserved portion of the RNA genetic element. These conserved features are critical for forming the ligand-binding pocket, and consequently mutations that alter these conserved features usually disrupt ligand binding. Therefore, if these diverse ligands are bound at the natural binding site in the orphan riboswitch, mutation of conserved nucleotides or structures should adversely affect binding. Indeed, mutations in the M1 variant of construct 108 *yjdF* that disrupt the base-pairing of P1 also reduce the apparent dissociation constant (*K*_D_) for proflavine, but do not greatly alter the overall pattern of bands produced by the in-line probing reaction. Likewise, the mutations in construct M2 that restore P1 base-pairing also restore binding affinity to near normal (Supplemental Fig. S2B). In contrast, single mutations that alter strictly conserved A (M3) or G (M4) nucleotides in the loop of P2 (Fig. 1B) eliminate binding of proflavine (Supplemental Fig. S2B) or riboflavin (Supplemental Fig. S3). These results suggest that the *yjdF* motif RNA employs conserved nucleotides and base-paired regions to form a binding pocket that is strikingly broad in its molecular recognition specificity.

For our second approach, a reporter-fusion construct was generated wherein DNA corresponding to the *yjdF* motif RNA from *B. subtilis* was fused in-frame to the *lacZ* gene from *Escherichia coli*. This construct was inserted into a plasmid vector and transformed into *B. subtilis* cells. The resulting reporter strain was used in a Biolog Phenotype MicroArray assay adapted to reveal gene expression changes caused by different growth media additives (see Materials and Methods). Of more than 600 different conditions or chemical agents tested, only five compounds present in the Biolog library selectively induced reporter gene expression driven by the *yjdF* motif RNA. Hits from this screen included chelerythrine, dequalinium, acriflavine, aminoacridine, and harmane (Supplemental Fig. S4), of which chelerythrine and harmane are natural alkaloids produced by some plants. These azaaromatic compounds all induced gene expression to a similar level as proflavine, which was included as a possible positive control based on its ability to be tightly bound by the RNA. These findings demonstrate that *yjdF* motif RNAs function as ligand-binding riboswitches, although the precise natural ligand cannot be determined from the current data.

Importantly, chelerythrine (Figs. 1C and 3), dequalinium, and harmane were further evaluated for binding by 108 *yjdF* RNA, and all three exhibit robust affinity (Supplemental Table S1) and trigger the same RNA structure changes that were observed for proflavine (Supplemental Fig. S2). Likewise, mutations M1, M2, and M3 in the 108 *yjdF* construct (Fig. 1B) exhibit the same trends for chelerythrine binding (Supplemental Fig. S5), as were observed for proflavine. Additional *yjdF* riboswitch aptamer representatives from *Bacillus endophyticus* (Supplemental Fig. S6), *Bacillus cereus* (Supplemental Fig. S7), *Ruminococcus gauvreauii* (Supplemental Fig. S8), and *Lactobacillus plantarum* (Supplemental Fig. S9) were tested, and all exhibit strong binding of chelerythrine. These results demonstrate that the binding characteristics exhibited by the *B. subtilis* RNA construct are likely to be representative of this entire orphan riboswitch class.

**FIGURE 2.**
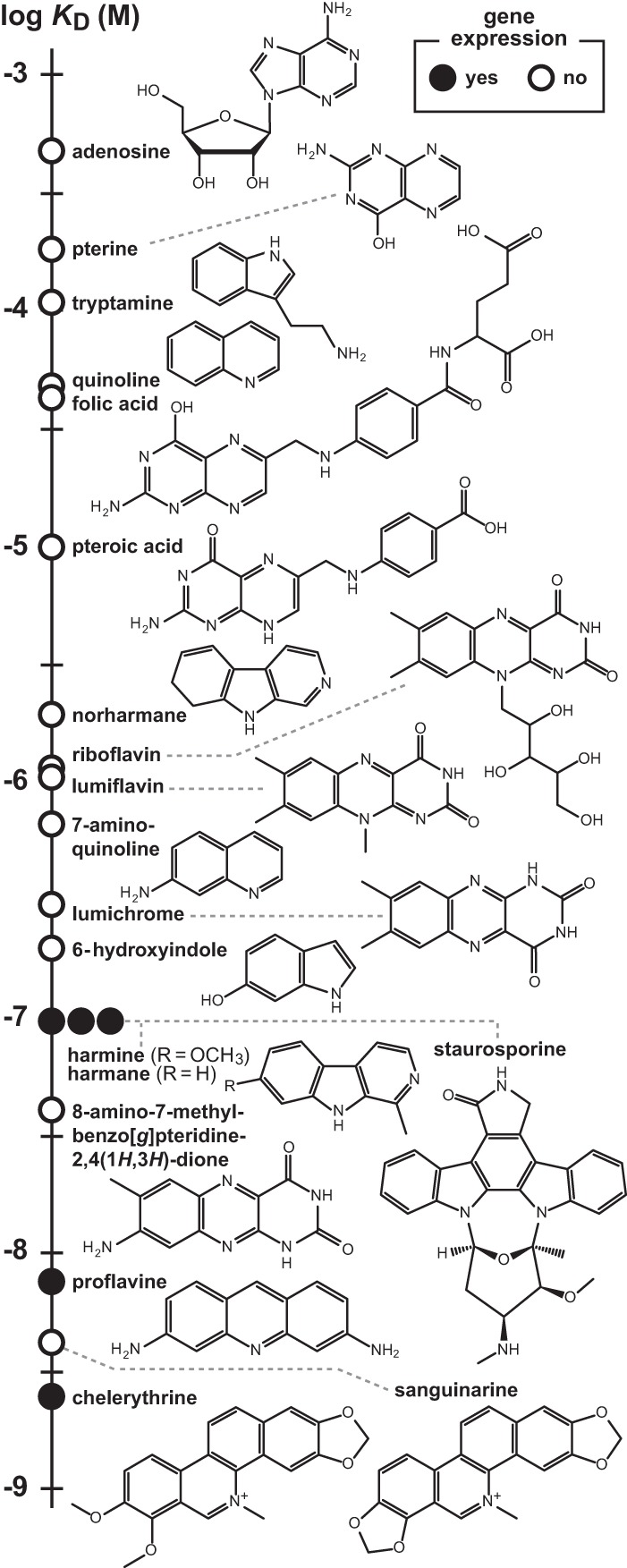
Binding affinities and gene expression activities of some compounds tested as candidate ligands for *yjdF* motif RNAs. Chemical structures are depicted for representative compounds tested for binding by the 108 *yjdF* RNA construct. Data points for compounds designating the *K*_D_ were established by in-line probing, and indicate whether the compound induces gene expression in the *B. subtilis yjdF* RNA-reporter strain.

Although each of these compounds identified by screening for gene expression induction are azaaromatic molecules, they otherwise have no distinctive chemical features in common with FMN and its various derivatives that we previously determined are bound by the 108 *yjdF* RNA construct. To determine whether additional diverse azaaromatic compounds are bound by the RNA, and to further establish a structure–activity relationship (SAR) between ligand binding and gene expression, we conducted additional binding and gene expression assays with a total of 130 compounds (Supplemental Table S1). A striking diversity of other compounds are bound by the 108 *yjdF* RNA construct (e.g., Fig. 2), demonstrating that the ligand-binding pocket formed by the RNA must not make an extensive use of hydrogen bonding to recognize a distinct pharmacophore. Rather, our results suggest that ligand binding might be driven mostly by other chemical interactions that can be formed more generally between RNA and azaaromatics. Consistent with this hypothesis is the fact that the product band patterns generated by in-line probing are virtually identical for the vast majority of compounds that are bound by the RNA. In other words, the RNA structural changes brought about by binding of these diverse compounds are remarkably similar, suggesting again that the binding pocket forms a similar shape regardless of the identity of the azaaromatic compound docked to the RNA.

### Nonspecific intercalation of RNA is not the mechanism for azaaromatic ligand binding and structural modulation of riboswitch aptamers

**FIGURE 3.**
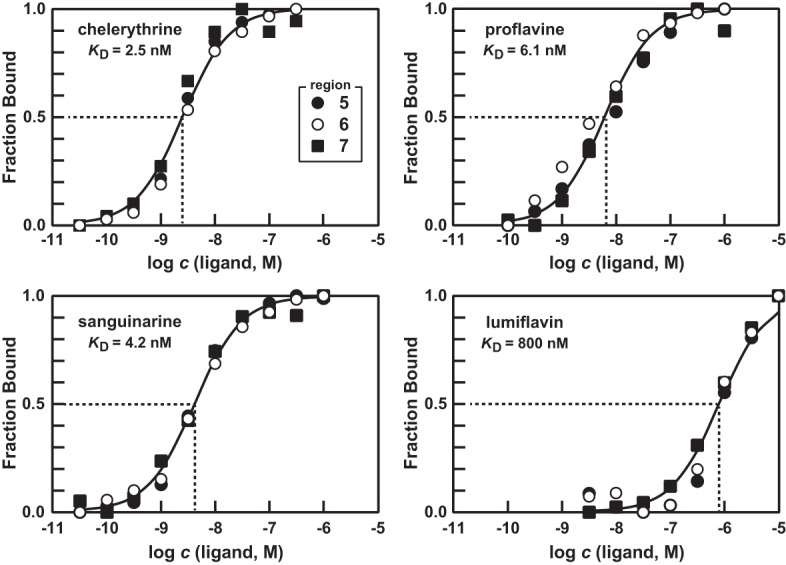
Binding curves for several azaaromatic ligands of *yjdF* RNA. Plots depict the estimated fraction of 108 *yjdF* RNAs bound to ligand versus the logarithm of the concentration (*c*) of ligand in molar units. Fraction bound values were estimated based on the extent of band intensity changes at regions 5, 6, and 7 as depicted in Figure 1B.

Some of the compounds that exhibit binding to the 108 *yjdF* RNA construct have long been known to function as nonspecific intercalators of DNA and RNA structures (e.g., see Finkelstein and Weinstein 1967). However, our data strongly indicates that the azaaromatics that are bound by *yjdF* RNAs are not broadly intercalating throughout the polymer, but are docking with one-to-one stoichiometry to a single, saturable binding site. For example, chelerythrine, its close analog sanguinarine, proflavine and the natural FMN degradation product lumiflavin all are bound by 108 *yjdF* RNA. Importantly, the binding data fit the standard dose–response curve with a Hill coefficient of 1, indicating that the binding of the ligand to the RNA is one-to-one. These results are consistent with the presence of a single saturable binding site that recognizes a single ligand molecule at any given time (Fig. 3).

Currently, there are no other riboswitch classes that are known to exhibit extremely broad ligand-binding characteristics by exploiting a single binding pocket. We speculated that FMN riboswitches (Mironov et al. 2002; Winkler et al. 2002) might be the best possible candidate for a class of RNAs that could simultaneously be broad in ligand specificity, yet use a single saturable binding site. FMN riboswitches are based on a consensus sequence and structure that is similar in complexity to *yjdF* motif RNAs (Supplemental Fig. S10A). Furthermore, FMN, or more precisely its riboflavin moiety, is a planar nitrogen-containing polycyclic compound with similarities to the azaaromatic compounds bound by *yjdF* RNAs. Therefore, the various molecules bound by *yjdF* RNAs might also bind well to FMN riboswitches, unless the FMN binding pocket has evolved to more strongly discriminate against diverse azaaromatic compounds.

Previous biochemical (Winkler et al. 2002) and structural (Serganov et al. 2009) analyses showed that members of this riboswitch class form a binding pocket that is very selective for FMN, and that strongly excludes most other flavin analogs due to precise hydrogen bonding and metal ion-mediated contacts between the riboswitch-binding site and FMN. To specifically determine whether FMN riboswitches have difficulty discriminating against other azaaromatic compounds, we conducted additional in-line probing assays with a 161-nt FMN binding construct derived from the *ribD* gene of *B. subtilis* (Supplemental Fig. S10B). Binding of FMN by the 161 *ribD* RNA induces a series of banding pattern changes with an apparent *K*_D_ of ∼1 nM (Supplemental Fig. S10C). Both this banding pattern and the *K*_D_ value are consistent with results obtained previously (Winkler et al. 2002). In contrast, neither proflavine nor chelerythrine can generate an in-line probing banding pattern with 161 *ribD* RNA that mimics the signal generated by FMN, even when tested at concentrations up to 10,000-fold higher than the *K*_D_ for the natural ligand. Indeed, the RNA construct begins to undergo random spontaneous cleavage when proflavine or chelerythrine are tested at 10 µM, suggesting that these compounds begin to bind to RNA nonspecifically before they are able to saturate the FMN-binding pocket.

Nonspecific intercalation is expected to cause general structural disruption of the aptamer, which should initially lead to increased spontaneous cleavage throughout the RNA polymer during in-line probing assays. Given that proflavine, chelerythrine, and other azaaromatic compounds do not induce spontaneous cleavage of RNA comprehensively along the length of the 108 *yjdF* RNA at concentrations needed to saturate the single-binding site, we conclude that *yjdF* motif RNAs might have naturally evolved to bind a broad diversity of azaaromatic compounds.

### Riboswitch function of *yjdF* motif RNAs is activated by certain azaaromatic compounds

**FIGURE 4.**
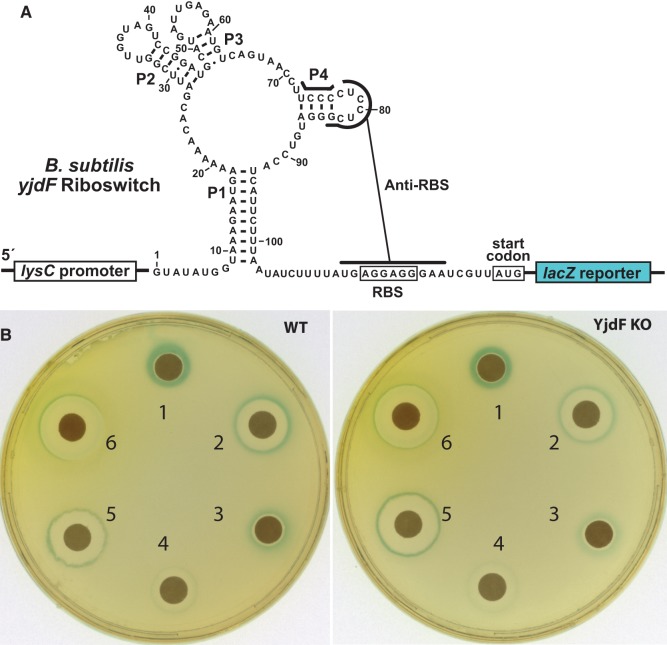
Riboswitch function by a *yjdF* RNA is activated by azaaromatic compounds. (*A*) Sequence and secondary structure of the regulatory region of the riboswitch–reporter construct generated by fusing the *yjdF* motif RNA from *B. subtilis* with the *lacZ* gene from *E. coli*. (*B*) Agar-diffusion assays wherein plates were inoculated with either WT or YjdF KO cells as indicated. Filter disks were infused with 10 µL of (1) staurosporine (10 mM), (2) harmane (100 mM), (3) harmine (100 mM), (4) norharmane (100 mM), (5) chelerythrine (1 mM), or (6) proflavine (10 mM).

The success of the Biolog screening campaign noted above demonstrated that some azaaromatic compounds can trigger activation of a reporter gene whose expression is regulated by the *yjdF* motif RNA from *B. subtilis*. When creating this reporter-fusion construct, we had speculated that the *yjdF* motif RNA from *B. subtilis* functions as a riboswitch by controlling access to the adjacent RBS through the formation of alternative base-paired structures (Weinberg et al. 2010). Specifically, 11 nucleotides that, in part, form the P4 stem–loop of the *yjdF* aptamer are complementary to 11 contiguous nucleotides encompassing the RBS for the *yjdF* coding region (Fig. 4A). When grafted to the coding region for *lacZ*, this *yjdF* motif, including the nucleotides that can form the alternative structure, forms the regulatory region for the β-galactosidase reporter gene assay. This same reporter construct was now used to determine the ability of various ligands to promote gene expression with either liquid or solid growth media in *B. subtilis* wild-type (WT) cells or in cells wherein the *yjdF* protein-coding region was deleted (YjdF KO).

Agar-diffusion assays revealed that most compounds that are poorly bound by the 108 *yjdF* RNA do not trigger expression of the reporter construct (Fig. 2; Supplemental Table S1). However, several compounds that bind with *K*_D_ values of 100 nM or better do activate gene expression driven by the riboswitch. For example, compounds such as staurosporine and harmane induce robust reporter gene expression (blue color) in cells that are closest to the filter disk that received the compound (Fig. 4B). At the highest concentrations nearest to the source of the diffusing compound, the blue color is absent, which reflects the fact that no cell growth occurs in this zone due to toxicity of the compound. Similar results are observed for chelerythrine and proflavine, which was expected since these compounds also activate gene expression in the Biolog screen. Moreover, given that WT and YjdF KO cells exhibit the same reporter gene expression results, the presence of the YjdF protein is not critical for riboswitch activation of gene expression with azaaromatic ligands.

**FIGURE 5.**
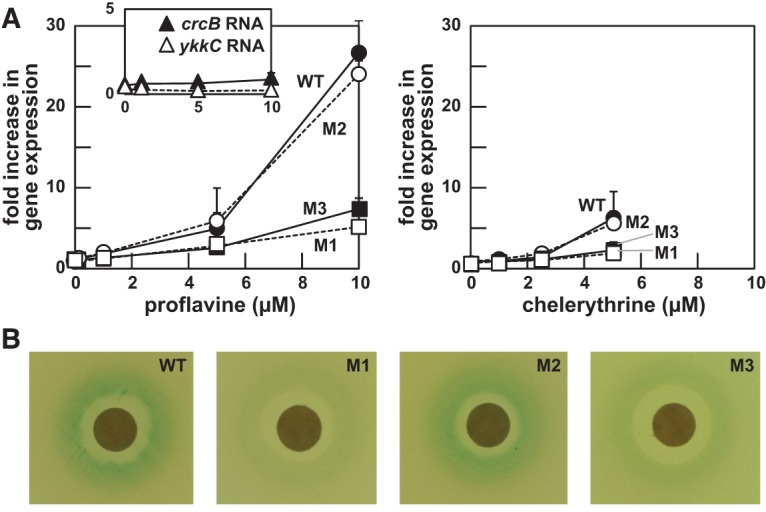
Gene expression mediated by an azaaromatic riboswitch. (*A*) Plots of β-galactosidase reporter gene expression data in WT *B. subtilis* cells carrying the *yjdF* RNA motif and flanking sequenced fused to the *lacZ* gene as depicted in Figure 4A. Cells carry either the WT *yjdF* motif RNA, or one of the mutations M1, M2, or M3 as depicted in Figure 1B. Cells are grown in the presence of proflavine (*left*) or chelerythrine (*right*). (*Inset*) Cells carrying riboswitch–reporter fusion constructs created by using the fluoride-responsive *crcB* riboswitch from *B. subtilis*, or the *ykkC* orphan riboswitch also from *B. subtilis*, were exposed to proflavine as a control. (*B*) Agar-diffusion assays using cells carrying the various reporter-fusion constructs as denoted and exposed to proflavine.

The importance of azaaromatic ligand binding is evident by reporter gene expression data generated by using both liquid and solid media. Reporter assays using liquid medium reveal that WT cells exhibit greater than a 25-fold increase in riboswitch-mediated gene expression upon the addition of 10 µM proflavine (Fig. 5A, left). In contrast, mutations M1 and M3 that disrupt conserved features of the aptamer domain yield markedly reduced response levels compared to the unmodified riboswitch. Restoration of the riboswitch structure in the M2 variant, which is known to restore ligand binding (Supplemental Figs. S2, S5), also restores near normal levels of proflavine-triggered gene activation. Importantly, proflavine does not induce reporter gene expression when unrelated riboswitch–reporter fusion constructs are tested that carry a *crcB* motif RNA (fluoride riboswitch) or a *ykkC* motif RNA (orphan riboswitch) (Fig. 5A, left, inset)

The various *yjdF* reporter constructs yield similar results with chelerythrine (Fig. 5A, right), although this compound is toxic to cells at 10 µM and therefore could not be tested at the same maximum concentration as used with proflavine. Curiously, although chelerythrine and sanguinarine have similar binding affinities (Fig. 2), sanguinarine was not observed to activate gene expression. There are several possible explanations for the failure of a strong ligand for a riboswitch to fail to activate gene expression. Perhaps the most likely explanation is that sanguinarine kills cells at a concentration below that needed to trigger riboswitch-mediated reporter gene expression, although many other explanations are possible. Similar gene expression patterns were observed for these molecules when tested on solid growth media (Fig. 5B), which again is consistent with our hypothesis that *yjdF* motif RNAs are riboswitches that can respond to certain azaaromatic compounds. Therefore, we have tentatively named members of this novel regulatory RNA class “azaaromatic riboswitches.”

### The natural ligand for azaaromatic riboswitches remains unknown

Riboswitches that sense toxic ligands, such as *S*-adenosylhomocysteine (SAH) (Wang et al. 2008), fluoride (Baker et al. 2012), or heavy metals (Furukawa et al. 2015) typically turn on the expression of genes whose protein products help cells overcome the toxic effects of the ligand. Notably, the riboswitch experimentally examined in this study also activates gene expression when certain azaaromatic compounds are present. Moreover, based on comparative sequence analysis (Weinberg et al. 2010), we predict that nearly all other azaaromatic riboswitches activate the production of YjdF proteins by permitting ribosome access to the RBS upon ligand binding. This fact suggests that the natural ligand (or the class of natural ligands) for azaaromatic riboswitches is toxic to cells, and that the YjdF protein has evolved to help cells overcome this toxicity.

Consistent with this hypothesis, azaaromatic compounds that trigger reporter gene expression are toxic to *B. subtilis* cells at high concentrations, as demonstrated by the lack of cell growth in close proximity to the filter disks in the agar plate assays (Fig. 4B). Unfortunately, although our findings reveal that numerous azaaromatic compounds are bound by *yjdF* motif RNAs and that some of these compounds with the highest affinity also activate gene expression, we cannot conclude that any of these molecules are representative of the natural ligand. If YjdF was expressed to overcome toxicity of any of these azaaromatic compounds tested, then differences in gene expression and toxicity should be apparent in the agar-diffusion assays comparing WT and YjdF KO cells unless there is a gene coding for a functionally redundant protein elsewhere in the genome. However, both the level of reporter gene expression and the extent of inhibition of cell growth are essentially identical between WT *B. subtilis* cells and cells carrying the YjdF KO (Fig. 4B).

We are unable to formulate a compelling structural model for the precise natural ligand by using the SAR data derived from our binding and gene expression assays and by using the SAR data derived from both the hits and the inactive compounds in the Biolog Phenotype MicroArray assay. Previous efforts with other riboswitch classes have successfully identified drug-like compounds that are bound nearly as tightly by the riboswitch as its natural ligand (Blount et al. 2007, 2015; Kim et al. 2009; Mulhbacher et al. 2010; Lünse et al. 2011). Although each of these compounds is a relatively close analog of the natural ligand, the *yjdF* motif RNAs examined in the current study bind to a large diversity of azaaromatic compounds. Therefore, based solely on our current data set, we have not been able to pinpoint the natural ligand or to identify a distinct pharmacophore that might be present in the natural ligand. Moreover, given the unprecedented chemical diversity of compounds that bind to and activate the riboswitch, we cannot rule out the possibility that azaaromatic riboswitches might have naturally evolved to recognize a large number of chemically diverse compounds, which would make them unique among the known classes of riboswitches.

To search for additional clues regarding the identity of the natural ligand for azaaromatic riboswitches, we reexamined the types of genes associated with the RNA motif and with the *yjdF* protein-coding gene (Supplemental Fig. S1). Only in very rare instances is a genetic element other than the coding region for YjdF located immediately downstream from azaaromatic riboswitches. Furthermore, there appears to be no relationship among these rare associations, and therefore we could derive no clues regarding the identity of the natural riboswitch ligand.

In contrast, we were intrigued by several genes sometimes associated with the YjdF coding region when the azaaromatic riboswitch was absent. Of 1826 YjdF coding regions examined, 1413 include an azaaromatic riboswitch in the 5′ UTR (Supplemental Fig. S1B). When the riboswitch was absent, the most common genetic element found, COG1695, is the coding region for PadR proteins. Members of this large family of gene regulation proteins are known to respond to a diverse array of phenolic acids, polycyclic aromatic hydrocarbons (PAHs), or azaaromatics (e.g., Gury et al. 2004; Madoori et al. 2009; Nguyen et al. 2011; Heravi et al. 2015). Using the Phyre2 program (Kelley et al. 2015), PadR proteins associated with azaaromatic riboswitches are predicted to form a structure very similar to the AphA protein from *Vibrio cholera* (De Silva et al. 2005). The AphA protein is structurally similar to the phenolic acid sensor MarR (Alekshun et al. 2001), which regulates a multiple antibiotic resistance operon in *Escherichia coli* (Sulavik et al. 1995).

Given these associations, we tested a series of phenolic acid compounds to determine whether they were tightly bound by the riboswitch and if they activated gene expression. Various compounds tested (Supplemental Fig. S11), such as cinnamic acid, caffeic acid, and acetylsalicylic acid were bound relatively weakly (*K*_D_ = 10 µM or poorer) compared to azaaromatics such as nalidixic acid (*K*_D_ = ∼1 µM). Moreover, none of these compounds triggered gene expression driven by the azaaromatic riboswitch reporter construct. Therefore, azaaromatic riboswitches are not likely to serve as sensors for natural phenolic acids. However, like the PadR family of transcription factors, the azaaromatic riboswitch class might be a versatile sensor for a class of natural toxic multi-ring compounds that have yet to be experimentally linked to this riboswitch class and to YjdF proteins.

### Conclusions

Advances in understanding bacterial gene control networks and mechanisms serve as strong motivation to uncover additional novel riboswitch classes. Each new riboswitch discovery reveals the super-regulon for its natural ligand among all the organisms that carry representatives of that riboswitch class. Genes that reside immediately downstream from a riboswitch, or that reside in an operon controlled by a riboswitch, are likely to be associated with the metabolic pathway, with the physiological adaptation system, or with the toxicity mitigation response system for the ligand that regulates gene expression. By examining the genetic contexts of all species that carry a particular riboswitch class, many genes (including those whose functions are unknown) can be linked to the processes that maintain homeostasis or detoxification of the ligand. For example, c-di-AMP has been implicated in signaling various responses to osmotic stress and cell wall remodeling (Block et al. 2010) via the genetic contexts of its riboswitch (Nelson et al. 2013). Similarly, c-AMP-GMP appears to be intimately involved in the regulation of bacterial exoelectrogenesis (Nelson et al. 2015). Without knowledge of these riboswitches, their ligands, and their gene associations, it would have been far more difficult to establish comprehensively the various roles performed by these signaling compounds.

In the current study, a combination of results derived from bioinformatics, in vitro binding assays, and in vivo gene expression assays demonstrate that *yjdF* motif RNAs function as ligand-responsive riboswitches. However, these data also reveal that members of this riboswitch class bind and respond to a surprisingly broad collection of ligands, which is unprecedented among the more than 30 other riboswitch classes that have been previously validated. On its own, the demonstration that this riboswitch class is unusually poor in its ability to discriminate against a broad range of ligands is not sufficient to conclude that RNA productively exploits this characteristic to detect a wide range of natural ligands. Similarly, our observation that a number of distinct azaaromatic compounds activate gene expression cannot be used as confirmation that the natural ligand or ligands for this riboswitch class can be classified as azaaromatic. It is possible to design and synthesize compounds that trick riboswitches into regulating gene expression by binding the natural ligand-binding pocket (Blount et al. 2007, 2015; Kim et al. 2009; Mulhbacher et al. 2010; Lünse et al. 2011; Howe et al. 2015). Therefore, the azaaromatic compounds that trigger gene expression in the current study might only be distal mimics of the natural target. In other words, azaaromatic compounds that trigger gene expression might reside in a unique region of chemical shape-space that is far from that occupied by the natural ligand. All these possibilities cause considerable uncertainty regarding the natural function of this riboswitch class.

However, the combination of our experimental findings, coupled with additional results derived from bioinformatics analyses, can best be explained currently by a hypothesis wherein azaaromatic riboswitches have evolved to serve as a general sensor of a class of natural azaaromatic ligands that are toxic to the host cells. Natural flavin compounds or their close derivatives are bound by *yjdF* motif RNAs with *K*_D_ values of 1 µM or better. Given that such compounds are widespread in biology, we considered very carefully the possibility that one or more flavin derivatives were the natural ligand. However, we believe it is unlikely that a compound (or class of compounds) closely related to FMN naturally triggers azaaromatic riboswitch function. Flavins or flavin-like compounds other than proflavine failed to trigger gene expression in our reporter assays, we only rarely observe *yjdF* motif RNAs near genes coding for flavin metabolism enzymes, and there are many other azaaromatic compounds that both bind more tightly and trigger gene expression.

Ligand binding is predicted to activate gene expression in the vast majority of examples, which is a hallmark of other riboswitches that bind toxic ligands. If true, the adjoining *yjdF* gene is likely to code for a protein that helps cells mitigate the toxic effect of the natural ligands. Consistent with this hypothesis is the fact that, when the azaaromatic riboswitch is absent, the most common gene located in its place codes for a PadR-like protein. PadR proteins are a large family of DNA-binding gene expression factors that are noteworthy because some have been found to be broad sensors for planar hydrophobic compounds (e.g., Gury et al. 2004; Madoori et al. 2009; Nguyen et al. 2011; Heravi et al. 2015) that are similar to azaaromatic compounds. It seems likely that the *padR* gene located immediately upstream of *yjdF* genes has adapted to sense the same general class of natural ligands that are sensed by the riboswitch, and that the proteins take over the regulatory role of the riboswitch in its absence.

Curiously, many of the species that carry azaaromatic riboswitches are known members of gut microbiomes, ranging from insects to humans. We considered the possibility that polycyclic antimicrobial compounds commonly found in the gut of many organisms might be sensed by azaaromatic riboswitches. For example, components of bile are polycyclic (but not azaaromatic) compounds that function both as emulsifiers for digestion of fats and also function as antibacterial compounds (Sung et al. 1993; Merritt and Donaldson 2009). However, bile extracted from bovine (Oxgal, Sigma-Aldrich), as well as the specific bile acid components taurodeoxycholic acid, chenodeoxycholic acid, and deoxycholic acid, all were bound poorly by 108 *yjdF* RNA construct as determined by in-line probing (Supplemental Table S1) and did not induce reporter gene expression (data not shown). We also tested a complex mixture of compounds derived from lignin, which is a polycyclic aromatic matrix whose components are known to have antimicrobial activity (Zemek et al. 1979; Dong et al. 2011) and might be present at high concentrations in the intestinal tract of organisms that consume plants. A lignin sample (Kraft, Sigma-Aldrich) does induce structural modulation of the 108 *yjdF* RNA construct in a manner consistent with azaaromatic ligands (Supplemental Fig. S12). However, we did not observe lignin-mediated activation of gene expression in the riboswitch–reporter fusion strain at concentrations up to 10 mg mL^−1^. Moreover, the YjdF protein KO strain also shows no special sensitivity to lignin in agar-diffusion assays.

Taken together, our findings demonstrate that azaaromatic riboswitches are able to broadly bind planar aromatic compounds, among which some of the most tightly bound compounds trigger gene expression in cells. However, the natural ligand or class of ligands remains elusive. Given that the riboswitch is unable to discriminate strongly against many azaaromatic compounds, and many of these ligands are not rendered less toxic by the YjdF protein, seeking the natural ligand by screening additional compounds for riboswitch binding and function is unlikely to convincingly reveal the ligand. Alternatively, it might be possible to conduct a chemical screen for compounds that are more toxic to cells lacking the YjdF protein compared to WT cells. Such compounds that also induce riboswitch-mediated gene expression would be excellent candidates for the natural ligand, and might reveal the identity of a class of compounds that are important for a large diversity of bacteria.

## MATERIALS AND METHODS

### Chemicals and DNA oligonucleotides

Chemical compounds and their sources are listed in Supplemental Table S1. All DNA oligonucleotides (primers and transcription templates) (Supplemental Table S2) were purchased from Sigma-Aldrich. [γ-^32^P] ATP was purchased from PerkinElmer.

### Plasmids and strains

Plasmid pDG1661 (Guérout-Fleury et al. 1996) and *B. subtilis* strain PY79 (BGSC 1A747) (Zeigler et al. 2008) were obtained from the Bacillus Genetic Stock Center (BGSC) at The Ohio State University.

### Bioinformatic analysis of *yjdF* representatives

Infernal (Nawrocki and Eddy 2013) was used to search for more *yjdF* examples from updated DNA sequence databases as described previously (Weinberg et al. 2015). Specifically, our analyses were conducted on sequences in the bacterial and archaeal section of RefSeq (Pruitt et al. 2007) version 56, and various environmental sequences collected from IMG/M (Markowitz et al. 2012), the Human Microbiome Project (Human Microbiome Project Consortium 2012), MG-RAST (Meyer et al. 2008), CAMERA (Sun et al. 2011), and GenBank (Benson et al. 2008). Sequence and secondary structure consensus models were constructed by using the software R2R (Weinberg and Breaker 2011).

### Preparation of RNA oligonucleotides

RNAs were prepared by in vitro transcription (Baker et al. 2012) using DNA templates generated by PCR using overlapping synthetic DNA oligonucleotides containing the promoter sequence for T7 RNA polymerase (Supplemental Table S2). Specifically, in vitro transcription reactions were performed using bacteriophage T7 RNA polymerase (T7 RNAP) in 80 mM *N*-(2-hydroxyethyl) piperazine-*N*′-(2-ethanesulfonic acid) (HEPES, pH 7.5 at 23°C), 40 mM dithiothreitol (DTT), 24 mM MgCl_2_, 2 mM spermidine, and 2 mM of each nucleoside 5′-triphosphate (NTP). RNA was purified using denaturing (8 M urea) 6% polyacrylamide gel electrophoresis (PAGE). Product bands corresponding in size to the desired products were visualized by UV shadowing, excised, and the RNA was eluted from the crushed gel slice using 10 mM Tris–HCl (pH 7.5 at 23°C), 200 mM NaCl, and 1 mM EDTA (pH 8.0). The RNA was precipitated with ethanol and pelleted by centrifugation.

To generate 5′ ^32^P-labeled RNAs, the 5′-terminal triphosphate was removed using alkaline phosphatase (Roche Diagnostics). The RNAs were then radiolabeled with [γ-^32^P] ATP (PerkinElmer) using T4 polynucleotide kinase (New England Biolabs). 5′ ^32^P-labeled RNAs were purified and isolated as described above.

### In-line probing of RNAs

In-line probing assays were performed as described previously (Regulski and Breaker 2008; Li and Breaker 2013). Briefly, 5′ ^32^P-labeled RNAs at a concentration of <5 nM were incubated with different concentrations of candidate ligands at 25°C for times ranging from 36 to 48 h in the presence of 100 mM KCl, 50 mM Tris–HCl (pH 8.3 at 23°C), and 20 mM MgCl_2_. RNA spontaneous cleavage products were resolved by denaturing 10% PAGE and imaged with a PhosphorImager (Molecular Dynamics). ImageQuant 5.1 was used to establish band intensities. Values for the apparent dissociation constants (*K*_D_) were determined as previously described (Baker et al. 2012).

### Design of reporter gene constructs

Plasmid pDG1661 was obtained from BGSC. An additional BamHI restriction site was introduced by site-directed mutagenesis using primers pDG1661-bamHI-F and pDG1661-bamHI-R (Supplemental Table S2). The region from −174 to +30 (relative to the *yjdF* gene translation start site) encompassing the *yjdF* motif RNA and the first 10 codons of the downstream ORF were amplified from *B. subtilis* genomic DNA using primers EcoRI-*lysC*-*yjdF*-vivo-F and *yjdF*-vivo-BamHI-R. The first primer was designed to introduce the *lysC* promoter from *E. coli* (Sudarsan et al. 2003), which promotes efficient RNA transcription and avoids any unknown layer of regulation that might be present in the natural *yjdF* promoter. The primers also carry restriction sites EcoRI and BamHI to facilitate cloning into pDG1661 by exploiting the EcoRI site and the introduced BamHI site. The final plasmid construct contains an in-frame translational fusion of *yjdF* RNA motif plus the first 10 codons of the yjdF ORF with the adjacent *lacZ* gene on the plasmid. The constructed plasmid is called pDG1661-*yjdF*-WT.

### Integration of the *yjdF motif*-*lacZ* reporter-fusion construct into *B. subtilis*

The plasmid pDG1661-*yjdF*-WT was transformed into *B. subtilis* strain PY79 (BGSC 1A747) (Zeigler et al. 2008) as described previously (Sudarsan et al. 2003). The transformation resulted in the integration of the fragment containing *lysC*-*yjdF*-*lacZ* into the genome of PY79 at the *amyE* locus, and successful transformants were identified by selecting colonies that are chloramphenicol resistant and spectinomycin sensitive (Sudarsan et al. 2003).

### *yjdF* gene knockout (KO) constructs

The procedure used for *yjdF* gene KO by homologous recombination using a KO cassette was similar to that described previously (Li et al. 2013). The KO cassette was composed of a 5′ flanking region, a spectinomycin resistance gene, and a 3′ flanking region. The 5′ flanking region (corresponding to nucleotide −928 to −127 relative to the *yjdF* start codon) and the 3′ flank fragment (corresponding to nucleotide +415 to +1223) were copied from *B. subtilis* genomic DNA by PCR using the appropriate primers (Supplemental Table S2). The spectinomycin resistance gene was copied from pDG1661 using primers Spec-F and Spec-R (Supplemental Table S2). The individual constructs were joined into one piece by using overlap extension PCR. The KO cassette was cloned into a pCR2.1-TOPO vector (Invitrogen), digested by BamHI and EcoRV restriction enzymes, and purified by agarose gel electrophoresis. The resulting fragment (∼1 µg) was used to transform *B. subtilis* strain PY79 to delete the DNA regions encoding both the *yjdF* motif RNA and its associated *yjdF* protein-coding gene (DUF2992) from the genome. Spectinomycin-resistant colonies were screened by PCR using the primers *yjdF*-check-F and *yjdF*-check-R (Supplemental Table S2) to confirm they contained the whole KO cassette in the genome, and that the deletion of the *yjdF* motif RNA and the DUF2992 gene had occurred using primers *yjdF*-ORF-F and *yjdF*-ORF-R. The KO strain was called *yjdF*-KO strain. Similarly, the KO cassette was used to knock out the *yjdF* RNA and DUF2992 from the *B. subtilis* strain that already contained the riboswitch-reporter fusion construct. The resulting strain was called *yjdF*-KO-*lacZ*-reporter strain.

### Agar diffusion and β-galactosidase assays

*Bacillus subtilis* strains carrying the riboswitch reporter constructs were grown for ∼16 h in LB liquid medium. Approximately 0.5 mL of each sample was spread on LB agar plates with 80 µg mL^−1^ X-gal and appropriate antibiotics. Autoclaved 6 mm diameter paper discs prepared from 0.35 mm thick pure cellulose chromatography paper (Fisher Scientific) were soaked with 10 µL of compounds at specific concentrations and transferred to the prepared agar plates. The plates were incubated at 37°C overnight to promote cell growth and then at 23°C for another 12–48 h prior to analysis.

### 4-Methylumbelliferyl β-d-galactopyranoside (4-MUG) assay for gene expression

Reporter gene assays were conducted using a method similar to that published previously (Vidal-Aroca et al. 2006; Nelson et al. 2013). Bacterial cell cultures were initiated from a single colony and grown overnight in 3 mL of medium (lysogeny broth, LB) with the appropriate antibiotics at 37°C with shaking. Absorbance at 595 nm (OD_595_) was measured and an inoculum was transferred to 2 mL of fresh LB with the appropriate antibiotics to yield an OD_595_ of 0.02. Test compounds were added as denoted for each experiment. The cultures were incubated at 30°C with shaking for 15 h. Eighty microliters of each sample (or fresh LB as a control) were added to three individual wells of a black Costar 96-well clear-bottom assay plate. OD_595_ values were established for each well by using an Infinite M200 PRO microplate reader (Tecan).

Subsequently, 80 µL of Z buffer (60 mM Na_2_HPO_4_, 40 mM NaH_2_PO_4_, 10 mM KCl, 1 mM MgSO_4_ [pH 7.0]) and 40 µL 1 mg mL^−1^ 4-Methylumbelliferyl β-d-galactopyranoside (Sigma-Aldrich) (dissolved in 50% DMSO and 50% deionized H_2_O) were added to each sample. The mixture was allowed to incubate at 23°C for 15 min, and 40 µL Na_2_CO_3_ was added to stop the reporter reaction. Fluorescence generated by β-galactosidase action on 4-MUG was measured by using an Infinite M200 PRO microplate reader with excitation at 360 nm and emission measurements at 460 nm. Arbitrary units of β-galactosidase activity (MUG units) were calculated as fluorescence intensity divided by total cell density (OD_595_).

## SUPPLEMENTAL MATERIAL

Supplemental material is available for this article.

## Supplementary Material

Supplemental Material

## References

[LIRNA054890C1] Alekshun MN, Levy SB, Mealy TR, Seaton BA, Head JF. 2001 The crystal structure of MarR, a regulator of multiple antibiotic resistance, at 2.3 A resolution. Nat Struct Biol 8: 710–714. 11473263 10.1038/90429

[LIRNA054890C2] Baker JL, Sudarsan N, Weinberg Z, Roth A, Stockbridge RB, Breaker RR. 2012 Widespread genetic switches and toxicity resistance proteins for fluoride. Science 335: 233–235. 22194412 10.1126/science.1215063 PMC4140402

[LIRNA054890C3] Barrick JE, Corbino KA, Winkler WC, Nahvi A, Mandal M, Collins J, Lee M, Roth A, Sudarsan N, Jona I, 2004 New RNA motifs suggest an expanded scope for riboswitches in bacterial genetic control. Proc Natl Acad Sci 101: 6421–6426. 15096624 10.1073/pnas.0308014101 PMC404060

[LIRNA054890C4] Benson DA, Karsch-Mizrachi I, Lipman DJ, Ostell J, Wheeler DL. 2008 GenBank. Nucleic Acids Res 36: D25–D30. 18073190 10.1093/nar/gkm929 PMC2238942

[LIRNA054890C5] Block KF, Hammond MC, Breaker RR. 2010 Evidence for widespread gene control function by the *ydaO* riboswitch candidate. J Bacteriol 192: 3983–3989. 20511502 10.1128/JB.00450-10 PMC2916388

[LIRNA054890C6] Blount KF, Wang JX, Lim J, Sudarsan N, Breaker RR. 2007 Antibacterial lysine analogs that target lysine riboswitches. Nat Chem Biol 3: 44–49. 17143270 10.1038/nchembio842

[LIRNA054890C7] Blount KF, Megyola C, Plummer M, Osterman D, O'Connell T, Aristoff P, Quinn C, Chrusciel RA, Poel TJ, Schostarez HJ, 2015 Novel riboswitch-binding flavin analog that protects mice against *Clostridium difficile* infection without inhibiting cecal flora. Antimicrob Agents Chemother 59: 5736–5746. 26169403 10.1128/AAC.01282-15 PMC4538501

[LIRNA054890C8] Breaker RR. 2011 Prospects for riboswitch discovery and analysis. Mol Cell 43: 867–879. 21925376 10.1016/j.molcel.2011.08.024 PMC4140403

[LIRNA054890C9] Breaker RR. 2012 Riboswitches and the RNA world. Cold Spring Harb Perspect Biol 4: a003566. 21106649 10.1101/cshperspect.a003566 PMC3281570

[LIRNA054890C10] Dambach M, Sandoval M, Updegrove TB, Anantharaman V, Aravind L, Waters LS, Storz G. 2015 The ubiquitous *yybP*-*ykoY* riboswitch is a manganese-responsive regulatory element. Mol Cell 57: 1099–1109. 25794618 10.1016/j.molcel.2015.01.035 PMC4376352

[LIRNA054890C11] De Silva RS, Kovacikova G, Lin W, Taylor RK, Skorupski K, Kull FJ. 2005 Crystal structure of the virulence gene activator AphA from *Vibrio cholerae* reveals it is a novel member of the winged helix transcription factor superfamily. J Biol Chem 280: 13779–13783. 15647287 10.1074/jbc.M413781200 PMC2652724

[LIRNA054890C12] Dong X, Dong M, Lu Y, Turley A, Jin T, Wu C. 2011 Antimicrobial and antioxidant activities of lignin from residue of corn stover to ethanol production. Ind Crops Prod 34: 1629–1634.

[LIRNA054890C13] Finkelstein T, Weinstein IB. 1967 Proflavine binding to transfer ribonucleic acid, synthetic ribonucleic acids, and deoxyribonucleic acid. J Biol Chem 242: 3763–3768. 5341263

[LIRNA054890C14] Furukawa K, Ramesh A, Zhou Z, Weinberg Z, Vallery T, Winkler WC, Breaker RR. 2015 Bacterial riboswitches cooperatively bind Ni^2+^ or Co^2+^ ions and control expression of heavy metal transporters. Mol Cell 57: 1088–1098. 25794617 10.1016/j.molcel.2015.02.009 PMC4667775

[LIRNA054890C15] Garst AD, Edwards AL, Batey RT. 2011 Riboswitches: structures and mechanisms. Cold Spring Harb Perspect Biol 3: a003533. 20943759 10.1101/cshperspect.a003533 PMC3098680

[LIRNA054890C16] Guérout-Fleury AM, Frandsen N, Stragier P. 1996 Plasmids for ectopic integration in *Bacillus subtilis*. Gene 180: 57–61. 8973347 10.1016/s0378-1119(96)00404-0

[LIRNA054890C17] Gury J, Barthelmebs L, Tran NP, Diviès C, Cavin JF. 2004 Cloning, deletion, and characterization of PadR, the transcriptional repressor of the phenolic acid decarboxylase-encoding *padA* gene of *Lactobacillus plantarum*. Appl Environ Microbiol 70: 2146–2153. 15066807 10.1128/AEM.70.4.2146-2153.2004 PMC383121

[LIRNA054890C18] Heravi KM, Lange J, Watzlawick H, Kalinowski J, Altenbuchner J. 2015 Transcriptional regulation of the vanillate utilization genes (*vanABK* operon) of *Corynebacterium glutamicum* by VanR, a PadR-like repressor. J Bacteriol 197: 959–972. 25535273 10.1128/JB.02431-14 PMC4325110

[LIRNA054890C19] Howe JA, Wang H, Fischmann TO, Balibar CJ, Xiao L, Galgoci AM, Malinverni JC, Mayhood T, Villafania A, Nahvi A, 2015 Selective small-molecule inhibition of an RNA structural element. Nature 526: 672–677. 26416753 10.1038/nature15542

[LIRNA054890C20] Huillet E, Velge P, Vallaeys T, Pardon P. 2006 LadR, a new PadR-related transcriptional regulator from *Listeria monocytogenes*, negatively regulates the expression of the multidrug efflux pump MdrL. FEMS Microbiol Lett 254: 87–94. 16451184 10.1111/j.1574-6968.2005.00014.x

[LIRNA054890C21] Human Microbiome Project Consortium. 2012 A framework for human microbiome research. Nature 486: 215–221. 22699610 10.1038/nature11209 PMC3377744

[LIRNA054890C22] Keefer LK, Johnson DE. 1970 Magnesium hydroxide as a thin-layer chromatographic adsorbent. II. A unique system for separating polynuclear azaaromatic compounds. J Chromatogr 47: 20–26. 5418120 10.1016/0021-9673(70)80004-8

[LIRNA054890C23] Kellenberger CA, Wilson SC, Hickey SF, Gonzalez TL, Su Y, Hallberg ZF, Brewer TF, Iavarone AT, Carlson HK, Hsieh YF, 2015 GEMM-I riboswitches from Geobacter sense the bacterial second messenger cyclic AMP-GMP. Proc Natl Acad Sci 112: 5383–5388. 25848022 10.1073/pnas.1419328112 PMC4418906

[LIRNA054890C24] Kelley LA, Mezulis S, Yates CM, Wass MN, Sternberg MJ. 2015 The Phyre2 web portal for protein modeling, prediction and analysis. Nat Protoc 10: 845–858. 25950237 10.1038/nprot.2015.053 PMC5298202

[LIRNA054890C25] Kim JN, Blount KF, Puskarz I, Lim J, Link KH, Breaker RR. 2009 Design and antimicrobial action of purine analogues that bind guanine riboswitches. ACS Chem Biol 4: 915–927. 19739679 10.1021/cb900146k PMC4140397

[LIRNA054890C26] Kim PB, Nelson JW, Breaker RR. 2015 An ancient riboswitch class in bacteria regulates purine biosynthesis and one-carbon metabolism. Mol Cell 57: 317–328. 25616067 10.1016/j.molcel.2015.01.001 PMC4538711

[LIRNA054890C27] Li S, Breaker RR. 2013 Eukaryotic TPP riboswitch regulation of alternative splicing involving long-distance base pairing. Nucleic Acids Res 41: 3022–3031. 23376932 10.1093/nar/gkt057 PMC3597705

[LIRNA054890C28] Li S, Smith KD, Davis JH, Gordon PB, Breaker RR, Strobel SA. 2013 Eukaryotic resistance to fluoride toxicity mediated by a widespread family of fluoride export proteins. Proc Natl Acad Sci 110: 19018–19023. 24173035 10.1073/pnas.1310439110 PMC3839697

[LIRNA054890C29] Lubelski J, De Jong A, van Merkerk R, Agustiandari H, Kuipers OP, Kok J, Driessen AJ. 2006 LmrCD is a major multidrug resistance transporter in *Lactococcus lactis*. Mol Microbiol 61: 771–781. 16879641 10.1111/j.1365-2958.2006.05267.x

[LIRNA054890C30] Lünse CE, Schmidt MS, Wittmann V, Mayer G. 2011 Carba-sugars activate the *glmS*-riboswitch of *Staphylococcus aureus*. ACS Chem Biol 6: 675–678. 21486059 10.1021/cb200016d

[LIRNA054890C31] Madoori PK, Aqustiandari H, Driessen AJ, Thunnissen AM. 2009 Structure of the transcriptional regulator LmrR and its mechanism of multidrug recognition. EMBO J 28: 156–166. 19096365 10.1038/emboj.2008.263 PMC2634732

[LIRNA054890C32] Markowitz VM, Chen IM, Chu K, Szeto E, Palaniappan K, Grechkin Y, Ratner A, Jacob B, Pati A, Huntemann M, 2012 IMG/M: the integrated metagenome data management and comparative analysis system. Nucleic Acids Res 40: D123–D129. 22086953 10.1093/nar/gkr975 PMC3245048

[LIRNA054890C33] McCown PJ, Liang JJ, Weinberg Z, Breaker RR. 2014 Structural, functional, and taxonomic diversity of three preQ_1_ riboswitch classes. Chem Biol 21: 880–889. 25036777 10.1016/j.chembiol.2014.05.015 PMC4145258

[LIRNA054890C34] Merritt ME, Donaldson JR. 2009 Effect of bile salts on the DNA and membrane integrity of enteric bacteria. J Med Microbiol 58: 1533–1541. 19762477 10.1099/jmm.0.014092-0

[LIRNA054890C35] Meyer F, Paarmann D, D'Souza M, Olson R, Glass EM, Kubal M, Paczian T, Rodriguez A, Stevens R, Wilke A, 2008 The metagenomics RAST server—a public resource for the automatic phylogenetic and functional analysis of metagenomes. BMC Bioinformatics 9: 386. 18803844 10.1186/1471-2105-9-386 PMC2563014

[LIRNA054890C65] Meyer MM, Hammond MC, Salinas Y, Roth A, Sudarsan N, Breaker RR. 2011 Challenges of ligand identification for riboswitch candidates. RNA Biol 8: 5–10. 21317561 10.4161/rna.8.1.13865 PMC3142362

[LIRNA054890C36] Mironov AS, Gusarov I, Rafikov R, Lopez LE, Shatalin K, Kreneva RA, Perumov DA, Nudler E. 2002 Sensing small molecules by nascent RNA: a mechanism to control transcription in bacteria. Cell 111: 747–756. 12464185 10.1016/s0092-8674(02)01134-0

[LIRNA054890C37] Mulhbacher J, Brouillette E, Allard M, Fortier LC, Malouin F, Lafontaine DA. 2010 Novel riboswitch ligand analogs as selective inhibitors of guanine-related metabolic pathways. PLoS Pathog 6: e1000865. 20421948 10.1371/journal.ppat.1000865 PMC2858708

[LIRNA054890C38] Nawrocki EP, Eddy SR. 2013 Infernal 1.1: 100-fold faster RNA homology searches. Bioinformatics 29: 2933–2935. 24008419 10.1093/bioinformatics/btt509 PMC3810854

[LIRNA054890C39] Nelson JW, Sudarsan N, Furukawa K, Weinberg Z, Wang JX, Breaker RR. 2013 Riboswitches in eubacteria sense the second messenger c-di-AMP. Nat Chem Biol 9: 834–839. 24141192 10.1038/nchembio.1363 PMC3830699

[LIRNA054890C40] Nelson JW, Sudarsan N, Phillips GE, Stav S, Lünse CE, McCown PJ, Breaker RR. 2015 Control of bacterial exoelectrogenesis by c-AMP-GMP. Proc Natl Acad Sci 112: 5389–5394. 25848023 10.1073/pnas.1419264112 PMC4418907

[LIRNA054890C41] Nguyen TK, Tran NP, Cavin JF. 2011 Genetic and biochemical analysis of PadR-*padC* promoter interactions during the phenolic acid stress response in *Bacillus subtilis* 168. J Bacteriol 193: 4180–4191. 21685295 10.1128/JB.00385-11 PMC3147689

[LIRNA054890C42] Östman CE, Colmsjö AL. 1987 Backflush HPLC for the isolation of polycyclic aromatic compounds—a comparative study. Chromatographia 23: 903–908.

[LIRNA054890C43] Price IR, Gaballa A, Ding F, Helmann JD, Ke A. 2015 Mn^2+^-sensing mechanisms of *yybP*-*ykoY* orphan riboswitches. Mol Cell 57: 1110–1123. 25794619 10.1016/j.molcel.2015.02.016 PMC4703321

[LIRNA054890C44] Pruitt KD, Tatusova T, Maglott DR. 2007 NCBI reference sequences (RefSeq): a curated non-redundant sequence database of genomes, transcripts and proteins. Nucleic Acids Res 35: D61–D65. 17130148 10.1093/nar/gkl842 PMC1716718

[LIRNA054890C45] Regulski EE, Breaker RR. 2008 In-line probing analysis of riboswitches. Methods Mol Biol 419: 53–67. 18369975 10.1007/978-1-59745-033-1_4

[LIRNA054890C46] Ren A, Wang XC, Kellenberger CA, Rajashankar KR, Jones RA, Hammond MC, Patel D. 2015 Structural basis for molecular discrimination by a 3′,3′-cGAMP sensing riboswitch. Cell Rep 11: 1–12. 25818298 10.1016/j.celrep.2015.03.004 PMC4732562

[LIRNA054890C48] Serganov A, Nudler E. 2013 A decade of riboswitches. Cell 152: 17–24. 23332744 10.1016/j.cell.2012.12.024 PMC4215550

[LIRNA054890C47] Serganov A, Patel DJ. 2012 Metabolite recognition principles and molecular mechanisms underlying riboswitch function. Annu Rev Biophys 41: 343–370. 22577823 10.1146/annurev-biophys-101211-113224 PMC4696762

[LIRNA054890C49] Serganov A, Huang L, Patel DJ. 2009 Coenzyme recognition and gene regulation by a flavin mononucleotide riboswitch. Nature 458: 233–237. 19169240 10.1038/nature07642 PMC3726715

[LIRNA054890C50] Soukup GA, Breaker RR. 1999 Relationship between internucleotide linkage geometry and the stability of RNA. RNA 5: 1308–1325. 10573122 10.1017/s1355838299990891 PMC1369853

[LIRNA054890C51] Sudarsan N, Wickiser JK, Nakamura S, Ebert MS, Breaker RR. 2003 An mRNA structure in bacteria that controls gene expression by binding lysine. Genes Dev 17: 2688–2697. 14597663 10.1101/gad.1140003 PMC280618

[LIRNA054890C52] Sulavik MC, Gambino LF, Miller PF. 1995 The MarR repressor of the multiple antibiotic resistance (mar) operon in *Escherichia coli*: prototypic member of a family of bacterial regulatory proteins involved in sensing phenolic compounds. Mol Med 1: 436–446. 8521301 PMC2230000

[LIRNA054890C53] Sun S, Chen J, Li W, Altintas I, Lin A, Peltier S, Stocks K, Allen EE, Ellisman M, Grethe J, 2011 Community cyberinfrastructure for advanced microbial ecology research and analysis: the CAMERA resource. Nucleic Acids Res 39: D546–D551. 21045053 10.1093/nar/gkq1102 PMC3013694

[LIRNA054890C54] Sung JY, Shaffer EA, Costerton JW. 1993 Antibacterial activity of bile salts against common biliary pathogens. Effects of hydrophobicity of the molecule and in the presence of phospholipids. Dig Dis Sci 38: 2104–2112. 8223087 10.1007/BF01297092

[LIRNA054890C55] Vidal-Aroca F, Giannattasio M, Brunelli E, Vezzoli A, Plevani P, Muzi-Falconi M, Bertoni G. 2006 One-step high-throughput assay for quantitative detection of β-galactosidase activity in intact Gram-negative bacteria, yeast, and mammalian cells. Biotechniques 40: 433–440. 16629389 10.2144/000112145

[LIRNA054890C56] Wang JX, Lee ER, Morales DR, Lim J, Breaker RR. 2008 Riboswitches that sense S-adenosylhomocysteine and activate genes involved in coenzyme recycling. Mol Cell 29: 691–702. 18374645 10.1016/j.molcel.2008.01.012 PMC2712820

[LIRNA054890C57] Weinberg Z, Breaker RR. 2011 R2R--software to speed the depiction of aesthetic consensus RNA secondary structures. BMC Bioinformatics 12: 3. 21205310 10.1186/1471-2105-12-3 PMC3023696

[LIRNA054890C58] Weinberg Z, Wang JX, Bogue J, Yang J, Corbino K, Moy RH, Breaker RR. 2010 Comparative genomics reveals 104 candidate structured RNAs from bacteria, archaea, and their metagenomes. Genome Biol 11: R31. 20230605 10.1186/gb-2010-11-3-r31 PMC2864571

[LIRNA054890C59] Weinberg Z, Kim PB, Chen TH, Li S, Harris KA, Lunse CE, Breaker RR. 2015 New classes of self-cleaving ribozymes revealed by comparative genomics analysis. Nat Chem Biol 11: 606–610. 26167874 10.1038/nchembio.1846 PMC4509812

[LIRNA054890C60] Winkler WC, Cohen-Chalamish S, Breaker RR. 2002 An mRNA structure that controls gene expression by binding FMN. Proc Natl Acad Sci 99: 15908–15913. 12456892 10.1073/pnas.212628899 PMC138538

[LIRNA054890C61] Zeigler DR, Prágai Z, Rodriguez S, Chevreux B, Muffler A, Albert T, Bai R, Wyss M, Perkins JB. 2008 The origins of 168, W23, and other *Bacillus subtilis* legacy strains. J Bacteriol 190: 6983–6995. 18723616 10.1128/JB.00722-08 PMC2580678

[LIRNA054890C62] Zemek J, Košíková B, Augustín J, Joniak D. 1979 Antibiotic properties of lignin components. Folia Microbiol (Praha) 24: 483–486. 389763 10.1007/BF02927180

[LIRNA054890C63] Zhang J, Lau MW, Ferré-D'Amaré AR. 2010 Ribozymes and riboswitches: modulation of RNA function by small molecules. Biochemistry 49: 9123–9131. 20931966 10.1021/bi1012645 PMC2965775

[LIRNA054890C64] Zheng G, Zhang Z, Lockman PR, Geldenhuys WJ, Allen DD, Dwoskin LP, Crooks PA. 2010 Bis-azaaromatic quaternary ammonium salts as ligands for the blood-brain barrier choline transporter. Bioorg Med Chem Lett 20: 3208–3210. 20462759 10.1016/j.bmcl.2010.04.098 PMC3725989

