# Supplementary material for: The yjdF riboswitch candidate regulates gene expression by binding diverse azaaromatic compounds
Source: RNA. 2016 Apr;22(4):530–41. doi: 10.1261/rna.054890.115 (PMC4793209; doi:10.1261/rna.054890.115)
Supplement: Supplemental Material [file supp_22_4_530__index.html]

The yjdF riboswitch candidate regulates gene expression by binding diverse azaaromatic compounds — The yjdF riboswitch candidate regulates gene expression by binding diverse azaaromatic compounds — Supplemental Material 

# The *yjdF* riboswitch candidate regulates gene expression by binding diverse azaaromatic compounds

## Supplemental Material

**Files in this Data Supplement:**

- Supp Material.docx
