# Supplementary material for: The yjdF riboswitch candidate regulates gene expression by binding diverse azaaromatic compounds
Source: RNA. 2016 Apr;22(4):530–41. doi: 10.1261/rna.054890.115 (PMC4793209; doi:10.1261/rna.054890.115)
Supplement: Supplemental Material [file supp_054890.115_SuppMaterial.docx]

**Supplemental Data**

**The *yjdF* riboswitch candidate regulates gene expression by binding diverse azaaromatic compounds**

SANSHU LI^1^, XUE YING HWANG^2^, SHIRA STAV^2^, and RONALD R. BREAKER^1,2,3^

^1^Howard Hughes Medical Institute, Yale University, Box 208103, New Haven, CT 06520-8103, USA

^2^Department of Molecular, Cellular and Developmental Biology, Yale University, Box 208103, New Haven, CT 06520-8103, USA

^3^Department of Molecular Biophysics and Biochemistry, Yale University, Box 208103, New Haven, CT 06520-8103, USA

Corresponding author: [ronald.breaker@yale.edu](mailto:ronald.breaker@yale.edu)

Dr. Ronald R. Breaker

Tel: (203) 432-9389

E-mail: [ronald.breaker@yale.edu](mailto:ronald.breaker@yale.edu)

**
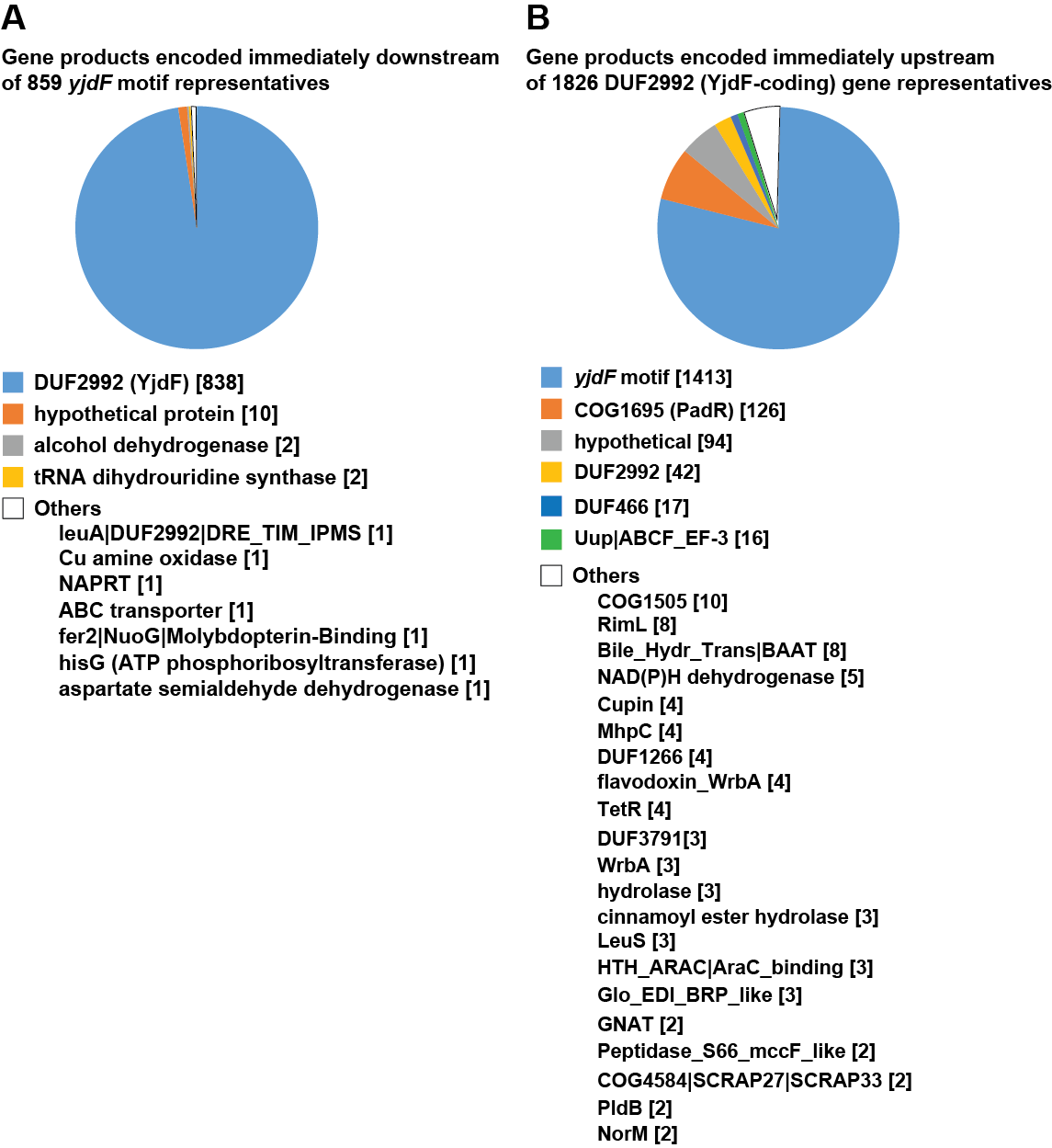
**

**FIGURE S1.** Genes associated with *yjdF* motif RNAs and YjdF protein-coding regions. (*A*) Graph representing the identities and distribution of genes located immediately downstream of the DNA template regions for 859 *yjdF* motif RNAs. (*B*) Graph representing the identities and distribution of genes or genetic elements located immediately upstream of 1826 YjdF-coding genes (not all associated with a *yjdF* motif RNA). YjdF is also called DUF2992. Note that the fourth most common genetic element associated with YjdF coding regions is another YjdF coding region, and therefore these genes occasionally reside in tandem. Note also that the dataset includes hits from all DNA database sources used, and some identical or near-identical examples from metagenomic sequence databases were not removed.

**
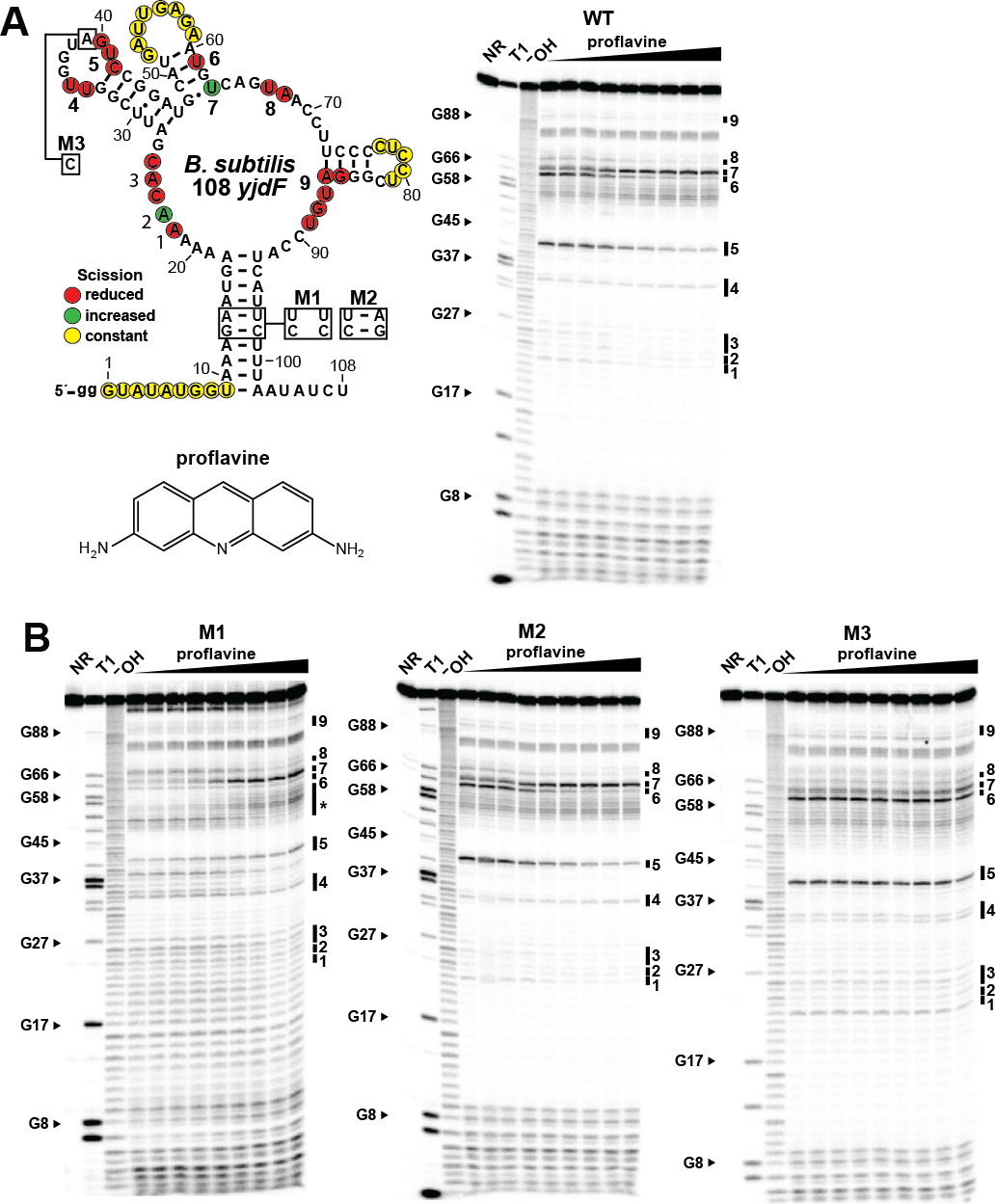
**

**FIGURE S2.** Binding characteristics of proflavine by the 108 *yjdF* RNA. (*A*) Wild-type (WT) 108 *yjdF* RNA construct from *B. subtilis* (left) binding to proflavine as determined by in-line probing (right). Proflavine concentrations range from 0 to 1 µM (differing by half-log increments). The dissociation constant (*K*_D_) determined by evaluating the fraction of RNAs bound to ligand (proportional to the extent of band modulation at each of the 9 designated sites) is presented in **Fig. 3**. Additional annotations are as described in the legend to **Fig. 1B** and **Fig. 1C**. (*B*) In-line probing data for proflavine binding by the mutant 108 *yjdF* RNA constructs M1 (left), M2 (center), and M3 (right). Additional annotations are as described in the legend to **Supplemental** **Fig. S2A** and **Fig. 1C**.


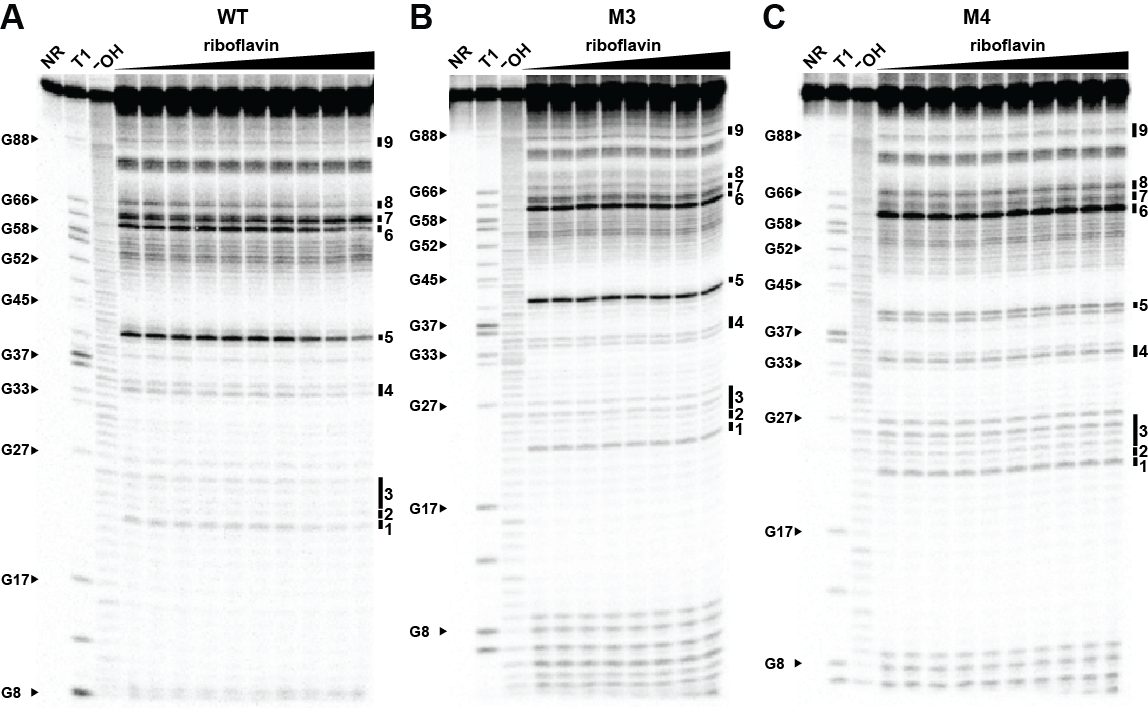


**FIGURE S3.** Binding characteristics of riboflavin by the 108 *yjdF* RNA and its mutants. (*A*) In-line probing results for the wild-type (WT) 108 *yjdF* RNA construct from *B. subtilis* binding to riboflavin. Riboflavin concentrations range from 0 to 10 µM. Additional annotations are as described in the legend to **Fig. 1B** and **Fig. 1C**. (*B*) In-line probing results for the 108 *yjdF* RNA construct M3 (**Fig. 1B**). Annotations are as described in A. (*C*) In-line probing results for the 108 *yjdF* RNA construct M4 (**Fig. 1B**). Annotations are as described in A. Note that structural modulation only occurs for the WT construct. The banding pattern induced by riboflavin binding is similar to that induced by proflavine binding, although the affinity for riboflavin is poorer (**Supplemental** **Table S1**).


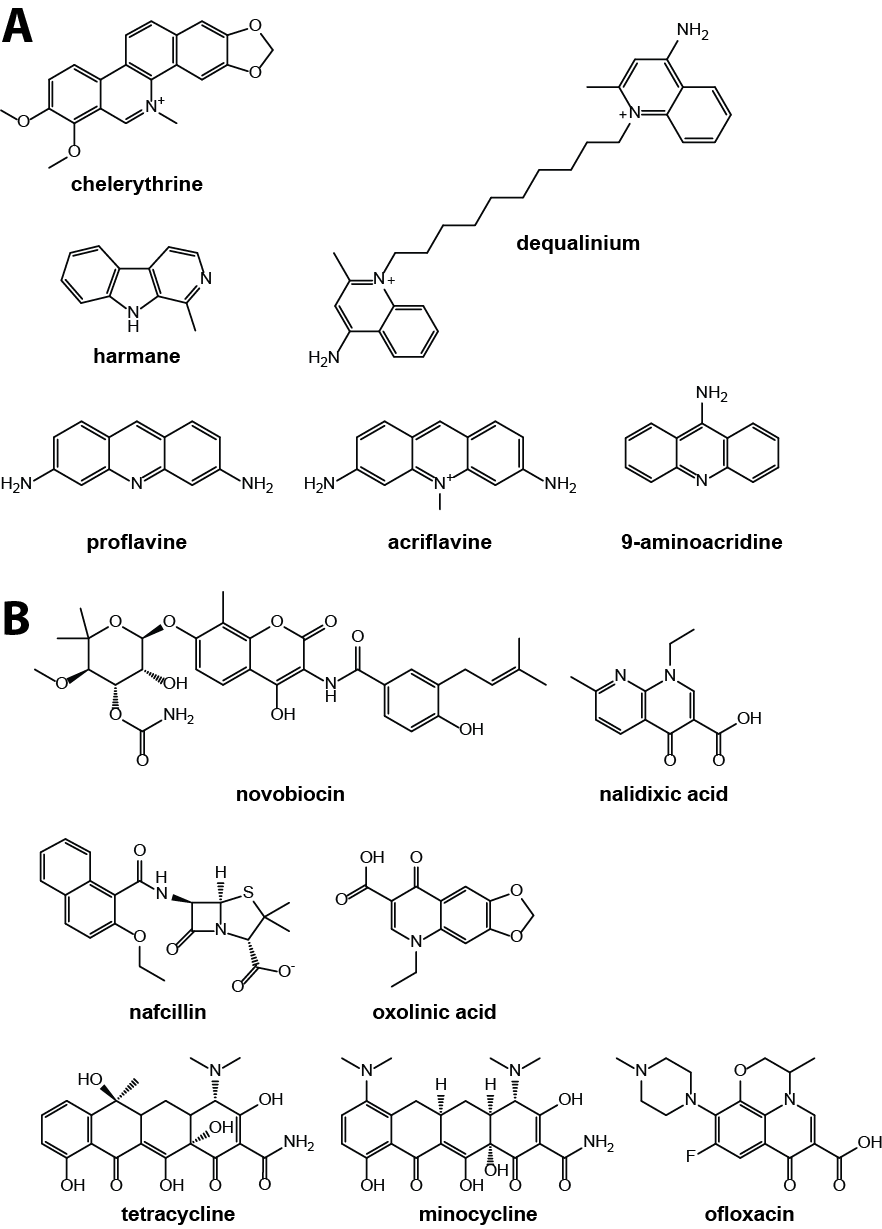


**FIGURE S4.** Some compounds present in the Biolog Phenotypic MicroArray screen. (*A*) Names and structures of compounds that activate gene expression in the Biolog screen. All compounds can be classified as azaaromatic. (*B*) Polycyclic compounds present in the Biolog screen that failed to activate gene expression.


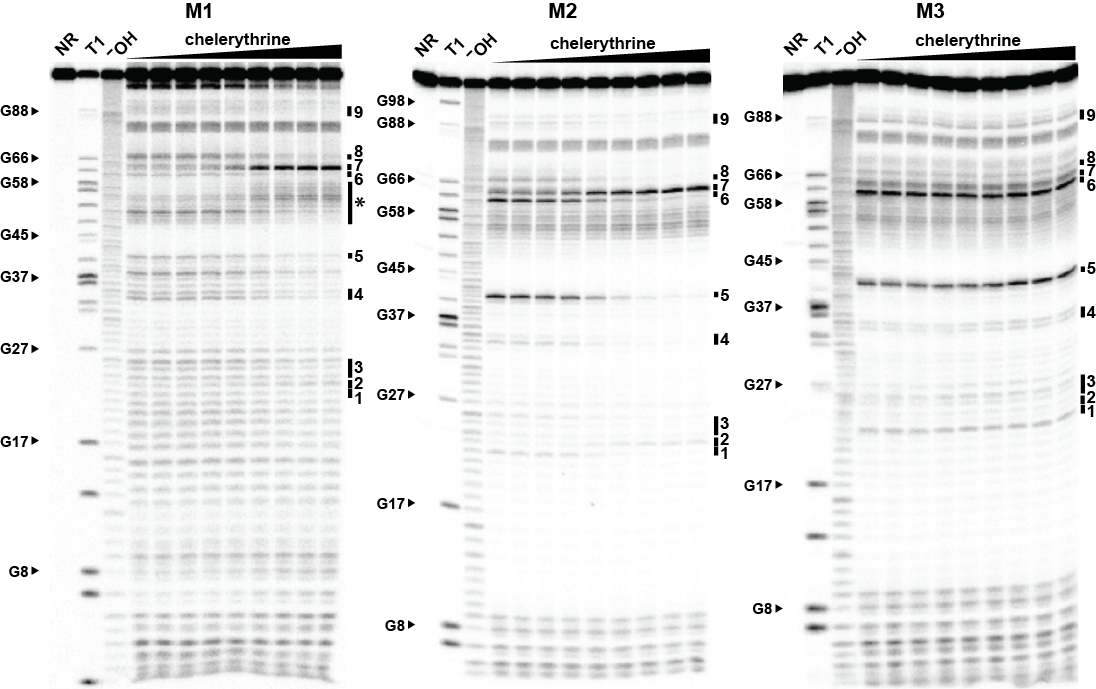


**FIGURE S5.** Binding characteristics of chelerythrine by the mutant 108 *yjdF* motif RNAs. In-line probing data for chelerythrine binding by the mutant 108 *yjdF* RNA constructs M1 (left), M2 (center), and M3 (right). Additional annotations are as described in the legend to **Supplemental** **Fig. S2A** and **Fig. 1C**.


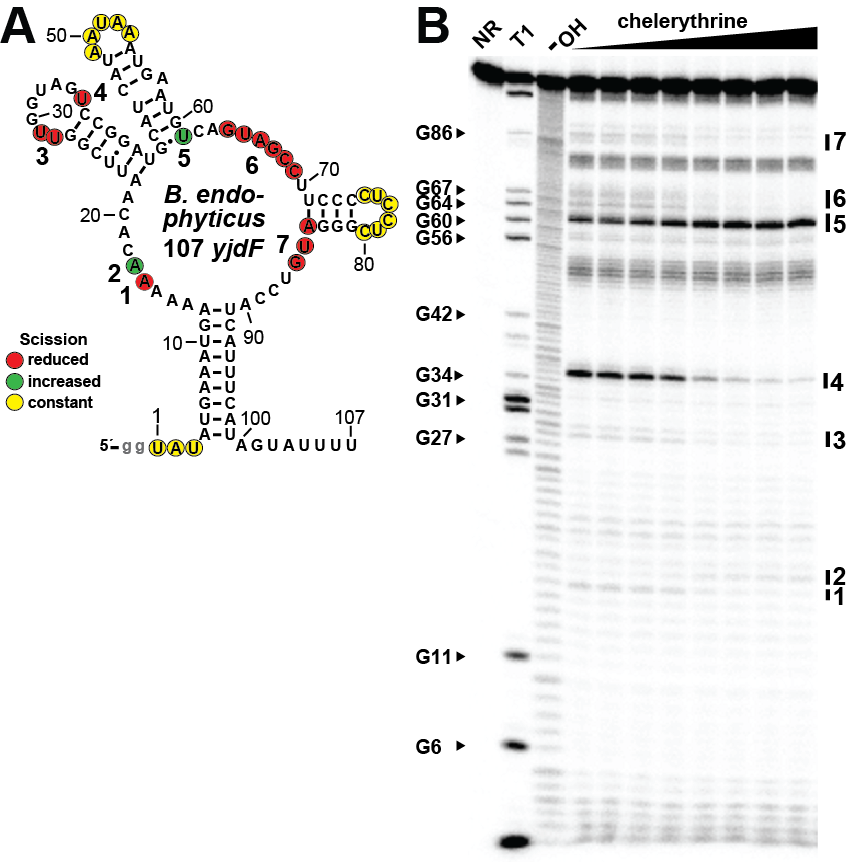


**FIGURE S6.** Binding characteristics of chelerythrine by the 107 *yjdF* RNA from *B. endophyticus.* (*A*) Sequence and secondary structure of the 107 *yjdF* RNA construct based on the 5ˊ UTR of the *yjdF* gene from *B. endophyticus*. Regions that undergo RNA strand scission as revealed by the in-line probing data depicted in B are identified with colored circles based on their characteristics. Seven regions that undergo increased or decreased scission are numbers 1 through 7. (*B*) In-line probing data for chelerythrine binding by the 107 *yjdF* RNA construct from *B. endophyticus*. Chelerythrine concentrations range from 0 to 1 µM. Additional annotations are as described in the legend to **Fig. 1C**.

**
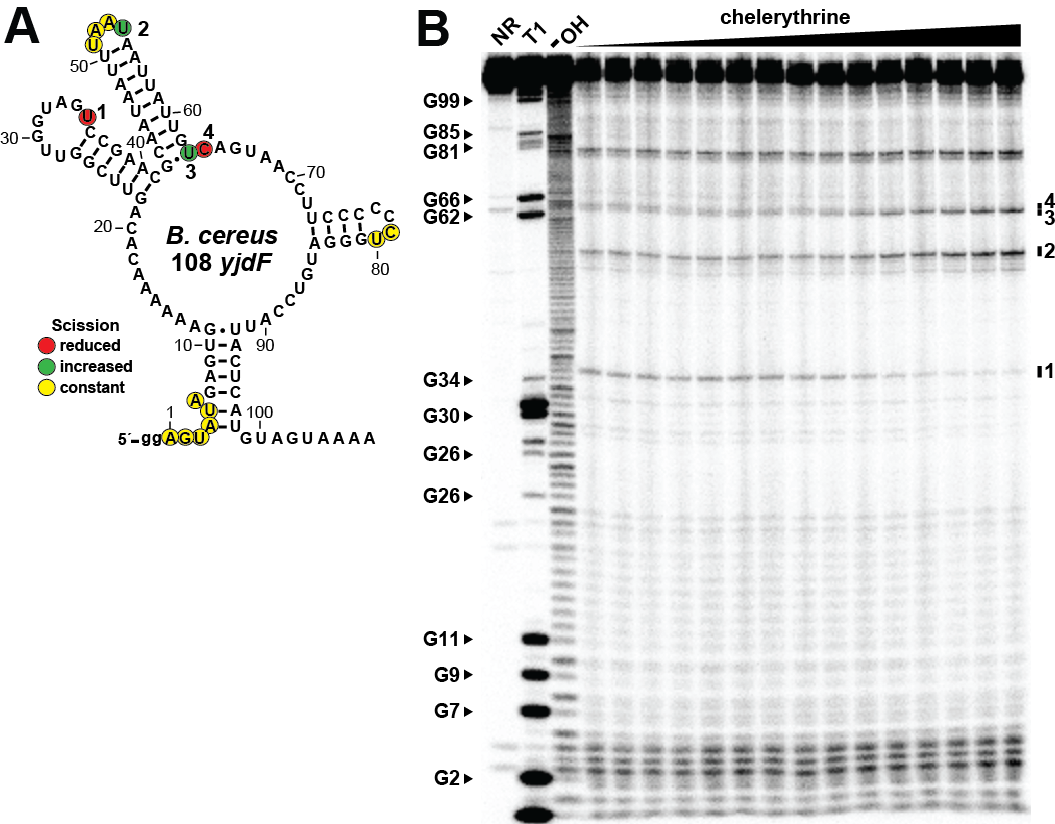
**

**FIGURE S7.** Binding characteristics of chelerythrine by the 108 *yjdF* RNA from *B. cereus.* (*A*) Sequence and secondary structure of the 108 *yjdF* RNA construct based on the 5ˊ UTR of the *yjdF* gene from *B. cereus*. Regions that undergo RNA strand scission as revealed by the in-line probing data depicted in B are identified with colored circles based on their characteristics. Four regions that undergo increased or decreased scission are numbers 1 through 4. (*B*) In-line probing data for chelerythrine binding by the 108 *yjdF* RNA construct from *B. cereus*. Chelerythrine concentrations range from 0 to 1 µM. Additional annotations are as described in the legend to **Fig. 1C**.


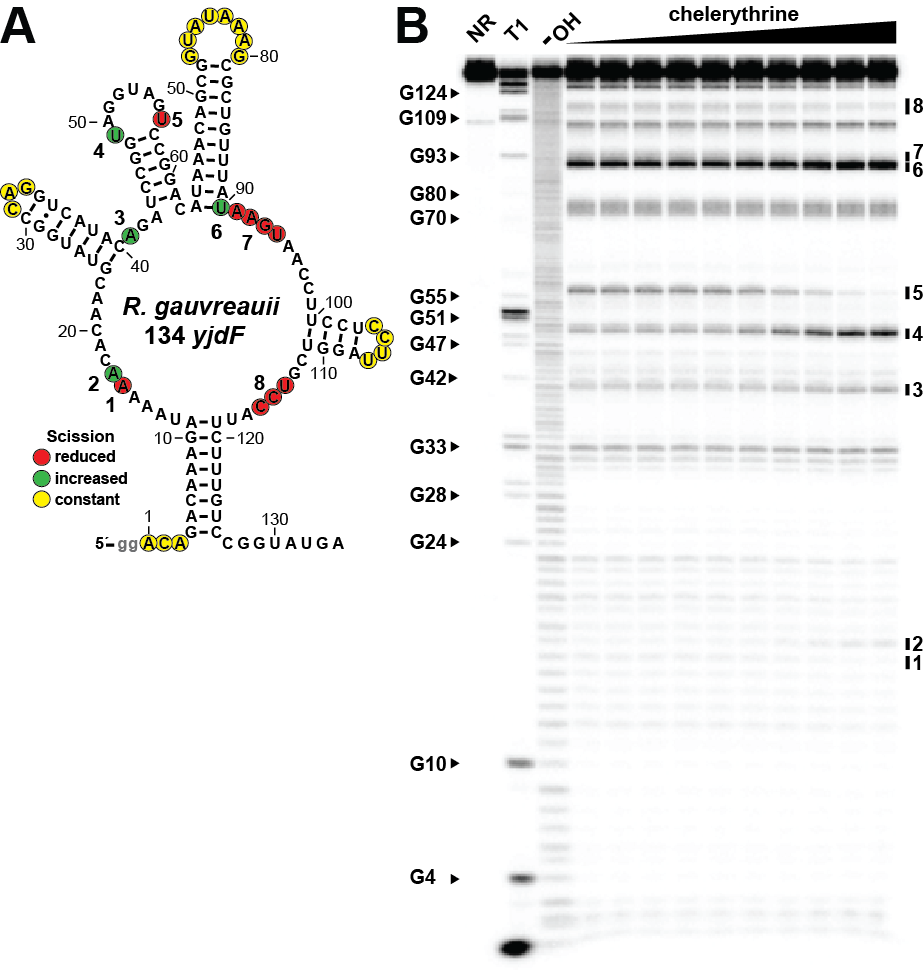


**FIGURE S8.** Binding characteristics of chelerythrine by the 134 *yjdF* RNA from *R. gauvreauii.* (*A*) Sequence and secondary structure of the 134 *yjdF* RNA construct based on the 5ˊ UTR of the *yjdF* gene from *R. gauvreauii*. Regions that undergo RNA strand scission as revealed by the in-line probing data depicted in B are identified with colored circles based on their characteristics. Eight regions that undergo increased or decreased scission are numbers 1 through 8. (*B*) In-line probing data for chelerythrine binding by the 134 *yjdF* RNA construct from *R. gauvreauii*. Chelerythrine concentrations range from 0 to 1 µM. Additional annotations are as described in the legend to **Fig. 1C**.


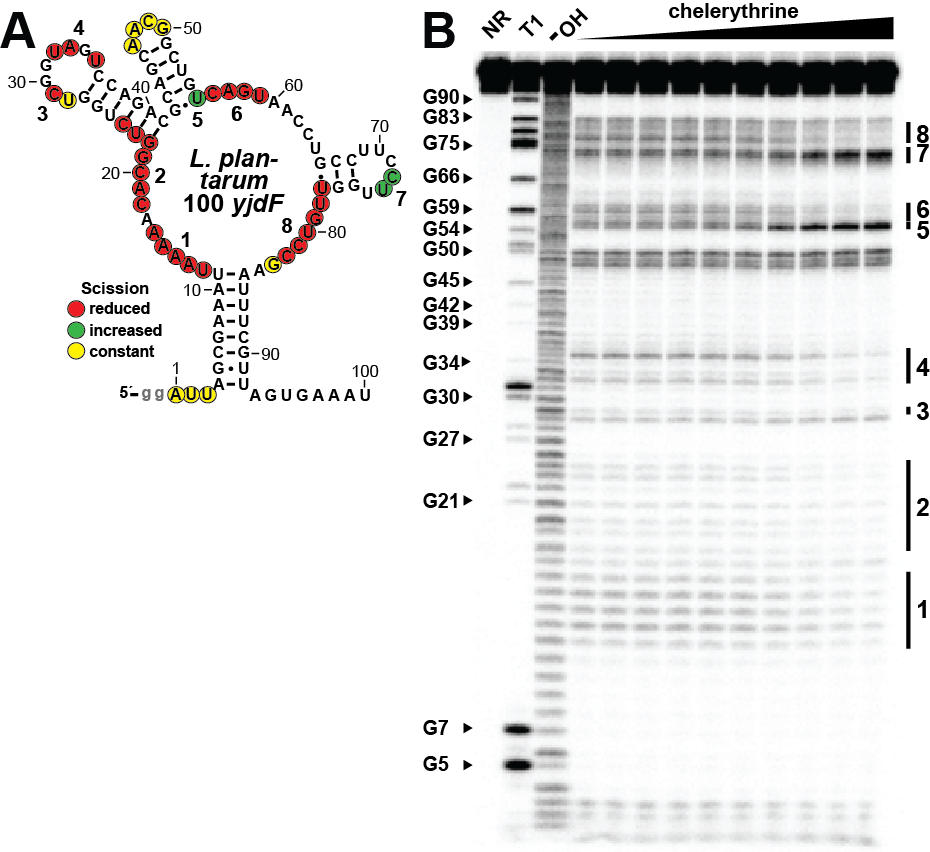


**FIGURE S9.** Binding characteristics of chelerythrine by the 100 *yjdF* RNA from *L. plantarum.* (*A*) Sequence and secondary structure of the 100 *yjdF* RNA construct based on the 5ˊ UTR of the *yjdF* gene from *L. plantarum*. Regions that undergo RNA strand scission as revealed by the in-line probing data depicted in B are identified with colored circles based on their characteristics. Eight regions that undergo increased or decreased scission are numbers 1 through 8. (*B*) In-line probing data for chelerythrine binding by the 100 *yjdF* RNA construct from *L. plantarum*. Chelerythrine concentrations range from 0 to 1 µM. Additional annotations are as described in the legend to **Fig. 1C**.


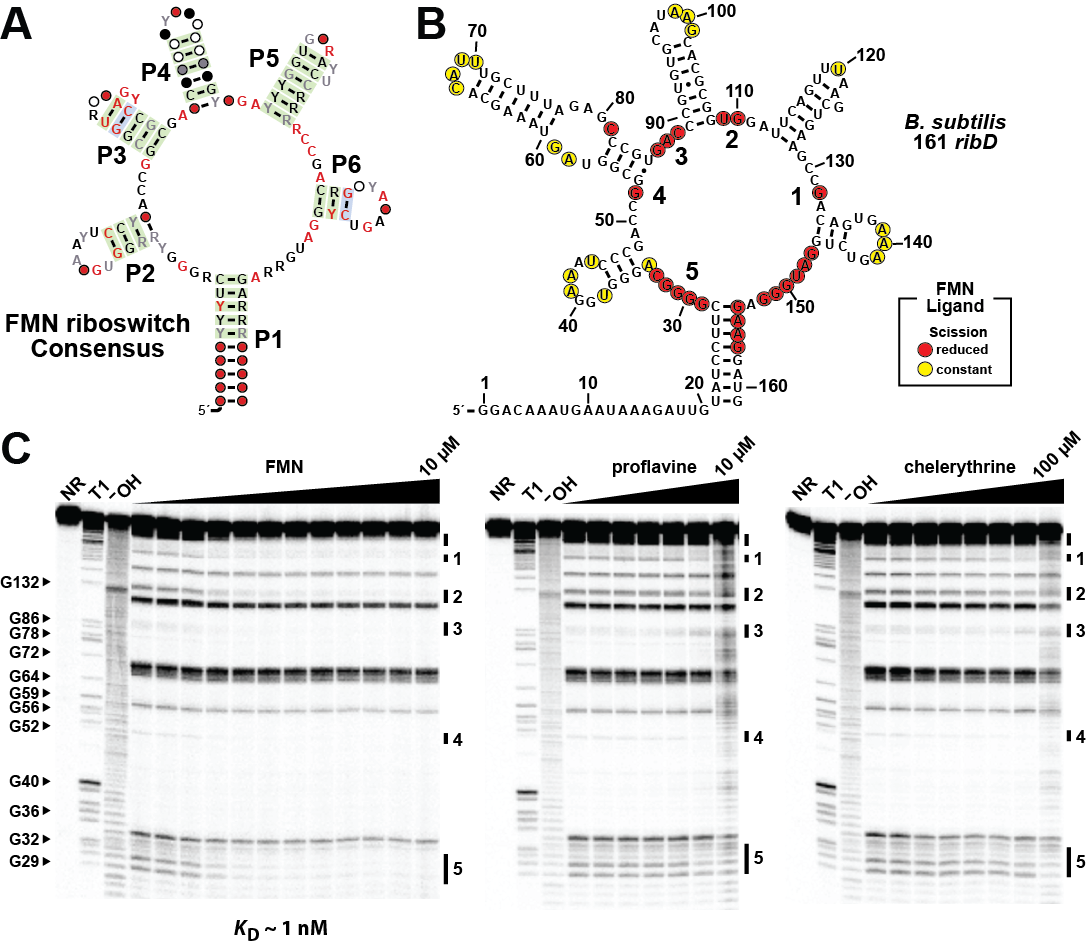


**FIGURE S10.** An FMN riboswitch aptamer strongly discriminates against other azaaromatic compounds. (*A*) The consensus sequence and structural model for FMN riboswitch aptamers. Members of this riboswitch class carry aptamers that have similar size and structural complexity compared to *yjdF* motif RNAs. Furthermore, FMN riboswitch aptamers naturally bind a ligand that has an azaaromatic moiety. Annotations are as described for **Fig. 1A**. (*B*) Sequence and secondary structure model for the FMN aptamer of the *ribD* riboswitch from *B. subtilis*. Annotations are as described for **Fig. 1B**. (*C*) In-line probing data for FMN, proflavine and chelerythrine binding by the 161 *ribD* RNA construct. Annotations are as described in the legend to **Supplemental** **Fig. S2A** and **Fig. 1C**.

**
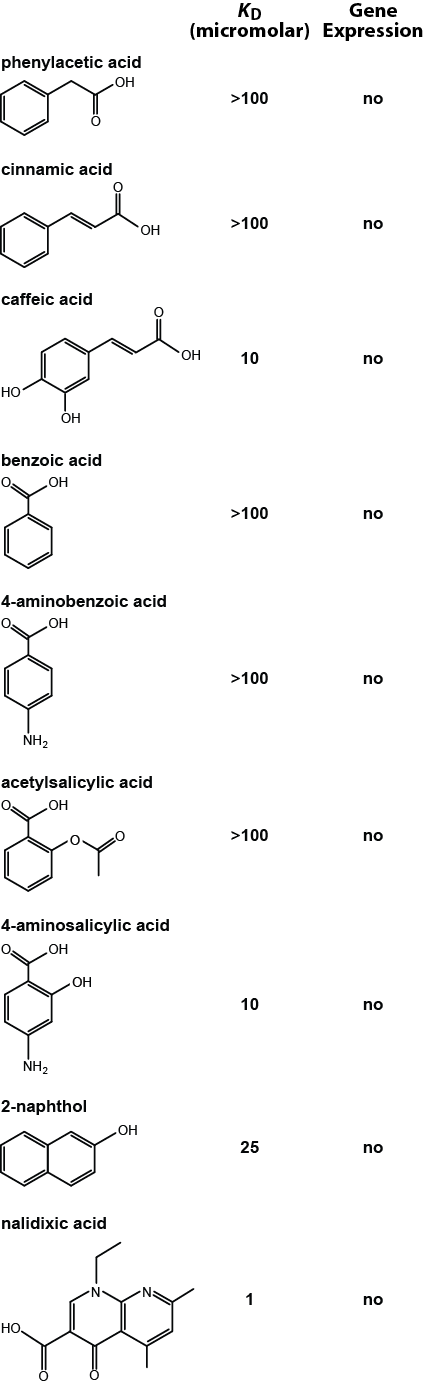
**

**FIGURE S11.** Phenolic acid compounds and derivatives examined for binding by the 108 *yjdF* RNA. *K*_D_ values were determined by in-line probing and gene expression functions were determined by agar diffusion assay with discs infused with 10 µL of a 10 mM solution of each compound. Note that the affinities of the phenolic acid compounds are all poorer than the PAH 2-naphthol and the azaaromatic nalidixic acid.

**
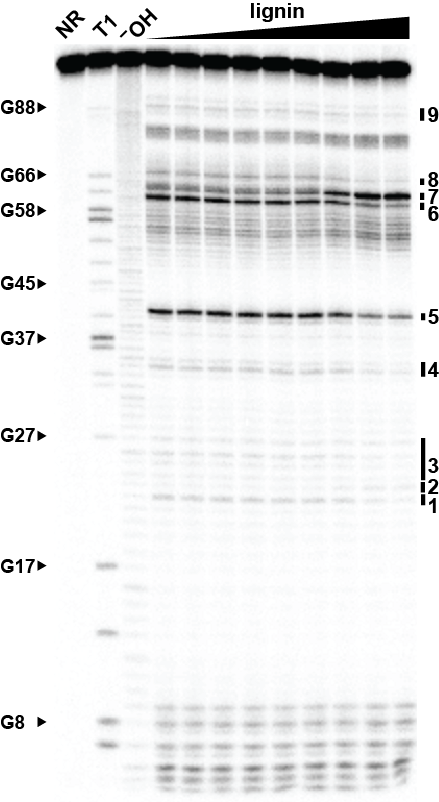
**

**FIGURE S12.** In-line probing assay of lignin binding by the 108 *yjdF* RNA. Note that this lignin sample is comprised of a collection of diverse lignin components including various phenolic acid monomers. The highest amount of this lignin mixture tested was 1 mg mL^-1^.

**TABLE S1.** Compounds used for evaluating *yjdF* motif RNAs. Note: *K*_D_ values were estimated from in-line probing data, and the compounds are presented from tightest binding to weakest binding.

| Compound Name | *K*_D_ (µM) | Source (Sigma-Aldrich unless otherwise noted) |
| --- | --- | --- |
| chelerythrine | 0.0024 |  |
| sanguinarine | 0.0043 | Cayman Chemical Company |
| proflavine (diaminoacridine) | 0.0061 |  |
| dequalinium | 0.01 |  |
| 8-amino-7-methylbenzo-pteridine-2,4(1H,3H)-dione | 0.04 | ChemBridge |
| daunorubicin | 0.05 |  |
| harmane | 0.1 |  |
| harmine | 0.1 | Cayman Chemical Company |
| staurosporine | 0.1 | LC Labs |
| (8-aminobenzo-pteridine-2,4 (3H,10H)-dione | 0.13 | ChemBridge |
| 6-hydroxyindole | 0.2 |  |
| 8-(dimethylamino)-benzo-pteridine-2,4(1H,3H)-dione | 0.3 |  |
| harmaline | 0.3 |  |
| lumichrome | 0.3 |  |
| 7-methyl-benzo-pteridine-2,4(1H,3H)-dione | 0.5 | ChemBridge |
| phenazine | 0.55 |  |
| 7-aminoquinoline | 0.6 |  |
| roseoflavin | 0.7 | MP Biomedicals |
| lumiflavin | 0.8 |  |
| 1-hydroxyPhenazine | 1.0 |  |
| nalidixic acid | 1.0 |  |
| pyocyanin | 1.0 |  |
| 7-methylquinoline-6-amine | 1.1 |  |
| alloxazine | 1.1 |  |
| flavin adenine dinucleotide (FAD) | 1.1 |  |
| riboflavin | 1.2 |  |
| 7-hydroxyquinoline | 1.6 |  |
| F0, (8-hydroxy-10-(2,3,4,5-tetrahydroxypentyl)pyrimido[4,5-b]quinoline-2,4(3H,10H)-dione) | 1.7 | Gift from Professor Thomas Carell, Ludwig Maximilian University of Munich |
| norharmane | 1.9 |  |
| flavin mononucleotide | 2.1 |  |
| 8-hydroxyquinoline | 3.6 |  |
| 6-hydroxyquinoline | 6.2 |  |
| pteroic acid | 9.9 |  |
| 2,6-dichloropurine | 10 | Oakwood Chemical |
| 2-amino-6-mercaptopurine | 10 |  |
| 4-aminosalicylate dihydrate | 10 |  |
| caffeic aicd | 10 |  |
| catechol | 10 |  |
| deoxycholic acid | 10 |  |
| preQ_0_ | 10 | Synthesized previously by our laboratory,  (Roth et al., 2007, Nat Struct Mol Biol, 14, 308-317) |
| 10-formyl tetrahydrofolate (THF) | 25 | Merck eprova AG |
| 2-naphthol | 25 |  |
| 5,10-methylene THF | 33 | Merck eprova AG |
| *N*,*N*-dimethylquinoline-6-amine | 35 | Vitas-M Laboratory, Ltd. |
| folic acid | 39 |  |
| guanosine-3',5'-cyclic monophosphorothioate (sp-isomer) | 44 | BioLog Life Science Institute |
| quinoline | 45 |  |
| guanosine-3',5'-cyclic monophosphate, acetoxymethyl ester | 82 | BioLog Life Science Institute |
| 5, 10-methenyl THF | 90 | Merck eprova AG |
| 6-methylamino-purine | 100 |  |
| trytamine | 109 |  |
| 8-hydroxyguanosine-3',5'-cyclic monophosphate | 111 | BioLog Life Science Institute |
| 7-methylquinoline | 122 |  |
| pterine | 180 |  |
| 2-aminopurine riboside-3',5'-cyclic monophosphate | 300 | BioLog Life Science Institute |
| *N*^2^-methylguanosine-3',5'-cyclic monophosphate (2-Me-cGMP) | ~ 1000 | BioLog Life Science Institute |
| adenosine 3'-monophosphate | ~100 |  |
| adenosine 3′,5′-cyclic monophosphate | ~100 |  |
| cytosine | ~1000 |  |
| thymine | ~1000 |  |
| 2,6-diaminopurine riboside | > 100 | MP Biomedicals |
| 2-aminopurine | > 100 |  |
| purine | > 100 |  |
| nicotinamide | > 1000 |  |
| nicotinamide mononucleotide | > 1000 |  |
| β-nicotinamide adenine dinucleotide phosphate (NADP) | > 1000 |  |
| 6,7 dimethyl-2,4-pteridinediol | > 3 | ChemBridge |
| hypoxanthine | > 3 |  |
| guanosine 5′-diphospho-D-mannose | >10 |  |
| cyclic di-AMP | >10 |  |
| tetracycline | >10 |  |
| trytophan | >10 |  |
| 10-formyl DHF | >100 | Merck eprova AG |
| 4-amino benzoic acid | >100 |  |
| 5-aminoimidazole-4-carboxamide ribonucleotide (AICAR) | >100 |  |
| 6,7-dimethyl-quinoline | >100 | Specs (www.specs.net) |
| acetylsalicylic acid | >100 |  |
| AP_4_, adenosine 5'-tetraphosphate | >100 | Jena Bioscience |
| benzoic acid (BA) | >100 |  |
| cinnamic acid | >100 |  |
| cyclic di-GMP | >100 | Axxora |
| cyclic AMP-GMP (c-AMP-GMP) | >100 | Axxora |
| erythromycin | >100 |  |
| guanine | >100 |  |
| pyrroloquinoline quinone | >100 |  |
| quinolinic acid | >100 |  |
| coenzyme B_12_ | >100 |  |
| 2-chloroinosine-3',5'-cyclic monophosphate | >1000 | BioLog Life Science Institute |
| 8-bromoguanosine-3',5'-cyclic monophosphate | >1000 | BioLog Life Science Institute |
| 8-Oxo-2'-deoxyguanosine-5'-triphosphate | >1000 | TriLink BioTechnologies |
| acetyl-CoA | >1000 |  |
| cytidine 2', 3'-cyclic monophosphate | >1000 |  |
| cytidine 3',5' cyclic monophosphate (cCMP) | >1000 |  |
| cytidine monophosphate (CMP) | >1000 |  |
| guanosine diphosphate | >1000 |  |
| inosine 3',5' cyclic monophosphate (cIMP) | >1000 |  |
| kanamycin | >1000 | AmericanBio |
| *N*-acetyl-D-glucosamine | >1000 |  |
| nicotinic acid | >1000 |  |
| nicotinic acid mononucleotide | >1000 |  |
| novobiocin | >1000 |  |
| phenylacetic acid | >100 |  |
| purine ribose-3',5'-cyclic monophosphate | >1000 |  |
| uracil | >1000 |  |
| uridine | >1000 |  |
| uridine 3', 5' cyclic monophophate (cUMP) | >1000 |  |
| xanthosine monophosphate (XMP) | >1000 |  |
| β-nicotinamide adenine dinucleotide (NAD) | >1000 |  |
| β-nicotinamide adenine dinucleotide phosphate, reduced (NADPH) | >1000 |  |
| chloramphenicol | 100-1000 |  |
| cytidine | 100-1000 |  |
| guanosine 5'-monophosphate | 100-1000 |  |
| 5-methyl DHF | 100-1000 | Merck eprova AG |
| 5-methyl THF | 100-1000 | Merck eprova AG |
| adenine | 100-1000 |  |
| adenosine | 100-1000 |  |
| Coenzyme Q_10_ | 100-1000 |  |
| guanosine | 100-1000 |  |
| *N*^1^-aminoguanosine-3',5'-cyclic monophosphate | 100-1000 | BioLog Life Science Institute |
| tetrahydrofolic acid (THF) | 100-1000 | Merck eprova AG |
| dihydrofolic acid (DHF) | 10-100 | Merck eprova AG |
| 2,6 diaminopurine | 10-100 |  |
| 5-Amino-4-imidazolecarboxamide (AICA) | 10-100 |  |
| 6-cyanopurine | 10-100 |  |
| 6-hydroxy-2-methylaminopurine | 10-100 |  |
| 6-mercaptopurine | 10-100 |  |
| lumazine | 300-1000 |  |
| folinic acid (5-formyl-THF) | 300-1000 |  |
| taurodeoxycholic acid | >100 |  |
| chenodeoxycholic acid | >100 |  |
| deoxycholic acid | >100 |  |

**TABLE S2.** Oligonucleotides used in this study.

| Primers | sequences | annotation |
| --- | --- | --- |
| *yjdF*-108-bsu-WT-t7-F | TAATACGACTCACTATAGgtatatgGTAAAGAATGAAAAAACACGATTCGGTTGGTAGTCCGGATGCATGATTGAG | Use Overlap PCR to synthesize 108-Bsu DNA |
| *yjdF*-108-bsu-R | AGATATTAAAGAATGATGGACATCCCGAGGAGGGGAAGGTTACTGACATTCTCAATCATGCATCCGGACTAC |  |
| *yjdF*-107-ben-t7-F | TAATACGACTCACTATAGgTATATGAAATGAAAAAACACAATTCGGTTGGTAGTCCGGATGCATCATAATAAATG | Use Overlap PCR to synthesize 107-Ben DNA |
| *yjdF*-107-ben-R | AAAATACTATGAAATGATGGACATCCCGAGGAGGGGAAGGCTACTGACATTCATTTATTATGATGCATCCGGACTACC |  |
| *yjdF*-108-bsu-M1-F | TAATACGACTCACTATAGgtatatgGTAAActATGAAAAAACACGATTCGGTTGGTAGTCCGGATGCATGATTGAG | Use this and *yjdF*-107-bsu-R to make 108-bsu-M1 DNA by overlap PCR |
| *yjdF*-108-bsu-M2-R | AGATATTAAActATGATGGACATCCCGAGGAGGGGAAGGTTACTGACATTCTCAATCATGCATCCGGACTAC | Use this and *yjdF*-107-bsu-M1-F to make 108-bsu-M2 DNA |
| *yjdF*-108-bsu-M3-F | TAATACGACTCACTATAGgtatatgGTAAAGAATGAAAAAACACGATTCGGTTGGTcGTCCGGATGCATGATTGAG | To synthesize 108-bsu-M3 DNA |
| *yjdF*-108-bsu-M3-R | AGATATTAAAGAATGATGGACATCCCGAGGAGGGGAAGGTTACTGACATTCTCAATCATGCATCCGGACgAC |  |
| 161-fmn-ribD-t7-F | TAATACGACTCACTATAGGACAAATGAATAAAGATTGTATCCTTCG | Use *B. subtilis* genomic DNA as template to synthesize 161-fmn-ribD DNA |
| 161-fmn-ribD-R | CATCCTTCTCCCATCCAGACTTTC |  |
| pDG1661-bamHI-F | ggttttcccggtcgGaTccgtaatcttacgtcag | to create an additional BamHI site on plasmid pDG1661. |
| pDG1661-bamHI-R | ctgacgtaagattacggAtCcgaccgggaaaacc |  |
| EcoRI-*lysC*-*yjdF*-vivo-F | aaaagaattcTgcaaaaataatgttgtccttttaaataagatctgataaaatgtgaactaatgttacactcttttctgtatatggtaaagaatg | Use PCR to make fragment containing *lysC* promoter and *yjdF* RNA motif to be fused in-frame with l*acZ* in the pDG1661 |
| *yjdF*-vivo-BamHI-R | ttttggatccctgaccatcgtagtaaatcgttaatttcataac |  |
| *yjdF*-ko-5F | gatgctgaccaggacttatctgttc | To make 5ʹ KO flank sequence with overlap with spec-fragment |
| *yjdF*-ko-5R | gtctacagattaataattattctttattatacagatcgatcccagaaaagagtgtaacatttcttctccaac |  |
| Spec-F | ggatcgatctgtataataaagaataattattaatctgtagac | To copy spectinomycin resistance gene from pDG1661 |
| Spec-R | ggatacttcccgtccgccag |  |
| *yjdF*-ko-3F | ctggcggacgggaagtatcctccaaacaaaagcaggcgcag | To make 3ʹ KO flank sequence with overlap with spec-fragment |
| *yjdF*-ko-3R | caaagagccatatcgctttatttgattgc |  |
| *yjdF*-KO-check-F | gctataccattccgtcacaacgaac | Use for PCR to check whether the KO cassette is integrated into genome.  WT strain would produce a 2555 bp fragment, while KO strains would have a 3192 bp fragment. |
| *yjdF*-KO-check-R | cttccaccgaagatcttggagacg |  |
| *yjdF*-ORF-F | aaattaacgatttactacgatggtcag | Use PCR to check *yjdF* open reading frame (ORF). WT would produce a 408 bp fragment, while KO would not. |
| *yjdF*-ORF-R | tttacctctgtgcttctttttcg |  |
